# Novel multimodal molecular imaging of Vitamin H (Biotin) transporter activity in the murine placenta

**DOI:** 10.1038/s41598-020-77704-9

**Published:** 2020-11-27

**Authors:** Noam Ben-Eliezer, Marina Lysenko, Inbal E. Bilton, Ofra Golani, Jennifer L. Bartels, Solana R. Fernandez, Tolulope A. Aweda, Nicholas A. Clanton, Rebecca Beacham, Suzanne E. Lapi, Joel R. Garbow, Michal Neeman

**Affiliations:** 1 Department of Biological Regulation, Weizmann Institute of Science, 7610001 Rehovot, Israel; 2 Department of Biomedical Engineering, Tel Aviv University, Tel Aviv, Israel; 3 Department of Veterinary Resources, Weizmann Institute of Science, 76100 Rehovot, Israel; 4 Life Sciences Core Facilities, Weizmann Institute of Science, 76100 Rehovot, Israel; 5 Department of Radiology, University of Alabama at Birmingham, Birmingham, AL USA; 6 Department of Chemistry, University of Texas at San Antonio, San Antonio, TX USA; 7 Biomedical MR Laboratory, Mallinckrodt Institute of Radiology, Washington University in St. Louis, St. Louis, MO USA

**Keywords:** Embryology, Reproductive biology, Preclinical research, Imaging, Fluorescence imaging, Magnetic resonance imaging, Molecular imaging, Positron-emission tomography, Molecular biophysics, Permeation and transport, Biomedical engineering

## Abstract

Vitamin H (biotin) is delivered to the fetus transplacentally by an active biotin-transport mechanism and is critical for fetal development. Our objective was to develop a comprehensive MRI technique for mapping biotin transporter activity in the murine placenta. Visualization of transporter activity can employ MRI’s unique T_2_*-dependent signal ‘off-switch’, which is triggered by transporter mediated aggregation of biotinylated contrast agent (b-BSA-Gd-DTPA). MRI data were collected from pregnant mice after administration of b-BSA-Gd-DTPA and analyzed using a new sub-voxel biophysical signal model. Validation experiments included competition with native biotin, comparative tests using PET, histology, and ICPMS. MRI signal was governed by binding, aggregation, and clearance of biotin (confirmed by histology). Signal dynamics reflected the placenta’s perfusion pattern modulated by biotin transporter activity and trophoblast mediated retention, and were in congruence with a three-compartment sub-voxel model. Pre-saturation of the transporters with free biotin suppressed b-BSA-Gd-DTPA uptake. The results were confirmed by PET, histology and ICPMS. The presented MRI-based platform allows to track activity of essential molecular transporters in the placenta, reflecting a transporter-mediated uptake, followed by retention and aggregation, and recycling associated with the large b-BSA-Gd-DTPA conjugate. The presented DCE-MRI technique can furthermore be used to map and characterize microstructural compartmentation and transporter activity without exposing the fetus to contrast media.

## Introduction

Vitamin H (biotin) is a water-soluble covalently bound coenzyme for carboxylases used in metabolic cell reactions, including fatty-acid synthesis and glucose and amino-acid metabolism^1^. Functional and immunological studies have shown that biotin transport is Na^+^-dependent in several organs, including intestine, kidney, and placenta^2,3^.

Biotin is delivered to the fetus transplacentally and is essential for normal fetal development^4^. Fetuses of biotin deficient mouse dams develop micrognathia, cleft palate, and micromyelia, while the frequency of malformations increases with decreasing biotin levels^5^. In humans, the concentration of biotin in the placenta is higher than in the maternal and fetal plasma, suggesting the existence of a selective biotin-transport and retention mechanism^5,6^. This transport is mediated by Na^+^-dependent multivitamin transporter (SMVT; product of the SLC5A6 gene) that is highly expressed in the human placenta^7^. Nonetheless, data on feto-maternal biotin levels in humans and in mice models are still scarce, conflicting, and possibly confounded by methodological problems^8^. A variety of ex vivo and post mortem methods have been used to study biotin transport in the placenta, including cell-based confocal microscopy^9^, gene manipulation^7,10^, and enzymatic activity assays of maternal and fetal samples from blood and urine^11^. Ex vivo analyses of histological sections provide detailed anatomical data, yet suffer from postexcision bias, and lack functional information. Consequently, development of new tools for noninvasive in vivo investigation of mouse placenta is essential for obtaining a real-time, functional understanding of vitamin transport in general, and biotin transport specifically.

In this study, dynamic contrast enhanced magnetic resonance imaging (DCE-MRI) was used to investigate biotin transporter activity and trophoblast cell retention in the mouse placenta. To achieve functional classification placental microscopic compartments, the macromolecular contrast agent biotin–bovine serum albumin–Gadolinium Diethylenetriaminepentaacetate (b-BSA-GdDTPA), which does not cross the maternal-fetus barrier, was employed, allowing selective probing of transporter mediated uptake and retention by placenta trophoblast cells with no transport to the fetal circulation. Based on a three-compartment mathematical placenta model, a new DCE-MRI signal-processing algorithm was introduced, yielding functional maps of biotin transport and uptake kinetics within different placental compartments, and evaluation of biotin transporter activity. Model predictions and MRI results were further validated using histology and PET imaging of biotin.

## Materials and methods

### Animals

Pregnant, female C57BL/6 mice were used in this study. Mice were housed with 12 h dark/light cycles and unlimited access to food and water. Mice underwent MRI scans on gestational day E14.5. All animal experiments were reviewed and approved by the Weizmann Institutional Animal Care and Use Committee (IACUC).

PET studies used pregnant, CD1 mice, purchased from Charles River, and delivered on gestational age of ~ E12-13. All animal studies were conducted in compliance with the guidelines for the care and use of research animals established by University of Alabama Birmingham Institutional Animal Care and Use Committee.

### Maternal–fetal biotin transport

To assess maternal–fetal biotin transport, biotin–Fluorescein (0.5 mg in PBS, Sigma-Aldrich) was administered to pregnant B6 mice at E14.5 (n = 4, 12 embryos) via tail vein. Mice were euthanized 60 min post injection. Placentae and embryos were fixed and sectioned serially. Histological sectioning was done for only a few consecutive slices, located at the mid sagittal section of the placenta. Placentas were fixed in Carnoy mixture, embedded in paraffin, and sectioned serially at 4-μm thickness.

Gadolinium content was independently determined by inductively coupled plasma mass spectrometry (ICPMS). Samples were homogenized with three equivalents of deionized water, and 100 µl of homogenate were lyophilized. Dry samples were digested with 200 µl of concentrated nitric acid (70%) overnight and heated in a dry bath for one hour at 90 °C. Standard, 10-ml samples were prepared with deionized water and analyzed by ICPMS (ELAN-6000 instrument; Perkin Elmer, Norwalk, CT). Statistical analysis of ICPMS data consisted of calculating the mean ± standard error of the mean (SEM). Differences between measurements were compared with repeated measures of two-way ANOVA using GraphPad Prism version 5.00 for Windows (GraphPad Software, San Diego, CA, USA).

### DCE-MRI imaging and analysis

MRI experiments were performed at 9.4T (BioSpec; Bruker, Germany). A group of mice (n = 10), were injected with b-BSA-GdDTPA using a bolus injection was via a tail vein catheter (Group A). A second *competition* group (n = 5) was injected with native biotin three minutes prior to administration of b-BSA-GdDTPA (group B). Dynamic T_1_-weighted 3D-GRE images were acquired at 20 time points post contrast injection for a total of 54 min.

During the course of these measurements, the concentration of the contrast agent changed continuously throughout all placental compartments. To assess contrast-agent perfusion kinetics, the mean signal intensity (SI) was recorded across the 20 time points and for each placenta. Due to the limited MRI frame rate (~ 3 min per scan), the concentration in each compartment was assumed to be stepwise constant for each individual scan. This resulted in a time-dependent signal, *S* (*n*) |_n=1…20_, for each voxel in the placenta.

**Figure 1:**
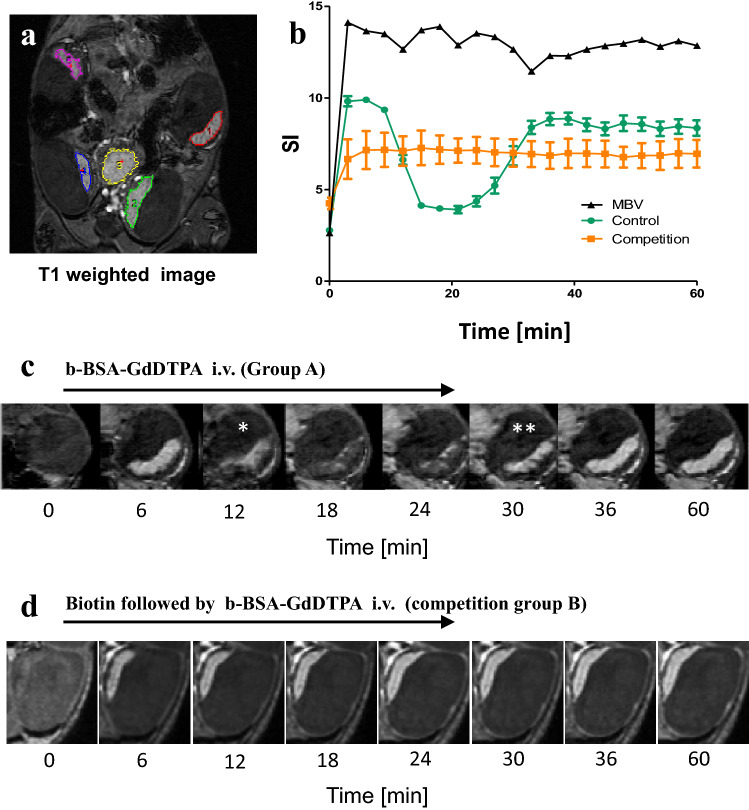
DCE-MRI analysis of placental signal kinetics in contrast-only and in biotin competition experiments. DCE-MRI experiments were performed on B6 (C57BL/6J) pregnant females on day E14.5 of gestation. Mice were divided into two groups. Group A was injected with contrast agent [b-BSA-GdDTPA, n = 6]; group B [n = 4] was injected with native biotin 3 min prior to contrast injection, in a “biotin competition” experiment. (**a**) T1-weighted 3D-GRE images of a representative mouse illustrating contrast-agent perfusion into the maternal arteries and uptake in the placentas. Colored regions mark the five placentae that are visible in the imaged slice. (**b**) Representative graph of the mean signal intensity (SI) vs. time in a placenta when injecting contrast agent only (green circles), biotin competition (orange squares), and in the vena cava (black triangles). Note the dip in the green signal curve, reflecting aggregation of contrast agent and drop in T_2_^*^ values (see "The effects of contrast agent aggregation" section). (**c**,**d**) Representative images at a chosen placenta showing signal change vs. time in contrast only injection (**c**), and biotin competition (**d**). *Initiation of Signal attenuation scan. **Initiation of signal recovery scan.

Placental regions of interest (ROIs) were marked using a semi-automated routine in Analyze Direct 11.0 (AnalyzeDirect Inc), initiated from manually planted seed points (Fig. 1a). ROIs were also drawn for the vena cava (maternal arterial input) and embryonal hearts. Three-dimensional data reconstruction was performed with AMIRA software (AMIRA, Mercury Computer Systems, Berlin, Germany) and Avizo software (Visualization Science Group, Burlington, MA) and movies displaying the DCE data were produced with Imaris Software (Bitplane AG, Zurich, Switzerland).

### Three-compartmental model of placental architecture

**Figure 2:**
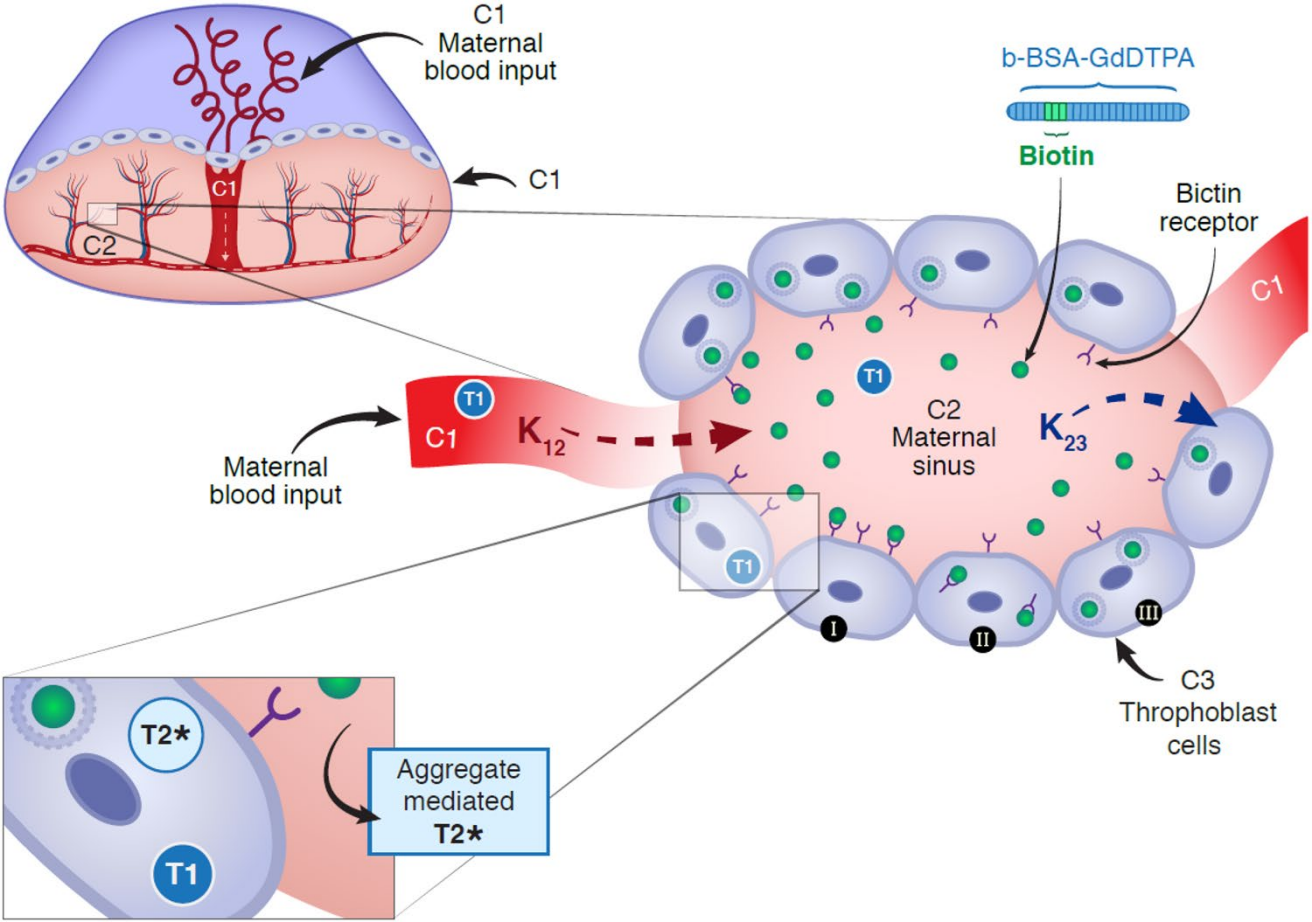
Placental three compartmental model. Placental internal structure is modeled as three main compartments. C1: the maternal arterial input; C2: the maternal intravascular compartment; and C3: the trophoblast cell compartment. k_12_ represents the rate of contrast agent flow from C1 into to C2 in the placenta and k_23_ represent the rate of contrast active uptake by the C3 compartment. Contrast material concentration within C1 was measured, and found to remain constant and equal to 104 µM ± 12.32 SD, N = 10. T1 and T_2_^*^ relaxation effects are shown in each compartment. While T_1_ relaxation is constant, the T_2_^*^ effect at C2 as a result of aggregate formation, is mediated via biotin transporters of the trophoblast cells lining the maternal sinuses, and the T_2_^*^ effect at C3 is mediated via active contrast-agent uptake by trophoblasts in the placental labyrinth. Later, retention in aggregate followed by recycling and dispersion is mediated via the C3 trophoblast cells in the labyrinth, which is concordant with the SI recovery phase in the MRI signal.

The placenta was modeled to consist of three microstructural sub-voxel compartments (Fig. 2): C1, large maternal blood pool, having a constant level of b-BSA-GdDTPA; C2, Spiral arteries perfusing the placenta; and C3, fetal trophoblast cells lining the labyrinth. In vivo DCE-MRI data cannot resolve these compartments, and were analyzed based on the assumption that each macroscopic voxel (size 150 × 150 × 625 µm^3^) contains contributions from C2 and C3, with C1 being easily detected and excluded from the processing. Following the bolus injection into C1, the contrast material is transported into C2 and C3 until a steady state is reached. The constant concentration in C1 was estimated to be approximately 100 mM based on the amount of contrast material injected divided by blood volume. Perfusion and inter-compartmental exchange evolved according to the following pair of differential equations:

1 $$\dot{c}_{2} = k_{12} \left( {c_{1} - c_{2} } \right) - k_{23} \left( {c_{2} - c_{3} } \right)$$

2 $$\dot{c}_{3} = k_{23} \left( {c_{2} - c_{3} } \right)$$

where *c*_1_, *c*_2_, and *c*_3_ are the concentrations within each compartment, $\dot{c}_{i}$ (i = 1, 2) represent the derivatives of each compartment’s concentration with respect to time, and *k*_12_ and *k*_23_ denote unidirectional inter-compartmental exchange rate constants. A detailed solution of these equations is given in the Appendix. The time dependent concentrations within each compartment were used to compute the relaxation rates *R*_1_(*t*) and *R*_2_(*t*) using typical relaxivity values^12^:

3 $$R_{1}^{(C2,C3)} (t) = c_{2,3} (t)\,[{\text{mM}}] \times 120\,[\sec^{ - 1} \,{\text{mM}}^{ - 1} ]$$

4 $$R_{2}^{(C2,C3)} (t) = c_{2,3} (t)\,[{\text{mM}}] \times 17\,[\sec^{ - 1} \,{\text{mM}}^{ - 1} ]$$

These values were then set into the steady-state signal equation for gradient echo (GRE) DCE-MRI^12^ yielding the time dependent signal arising from each voxel in the MRI images

5 $$S_{spoiled - GRE} (t) = \frac{{M_{0} \sin (\theta ) \cdot \left( {1 - e^{{TR \cdot R_{1} (t)}} } \right)}}{{1 - \cos (\theta )e^{{TR \cdot R_{1} (t)}} }}e^{{ - TE \cdot R_{2}^{*} }} .$$

Here $\theta$ denotes the excitation flip angle, *M*_0_ the proton density weighted by the receiver coil sensitivity profile, TR/TE the repetition/echo time, and $T_{2}^{*}$ the local field inhomogeneity.

### The effects of contrast agent aggregation

**Figure 3:**
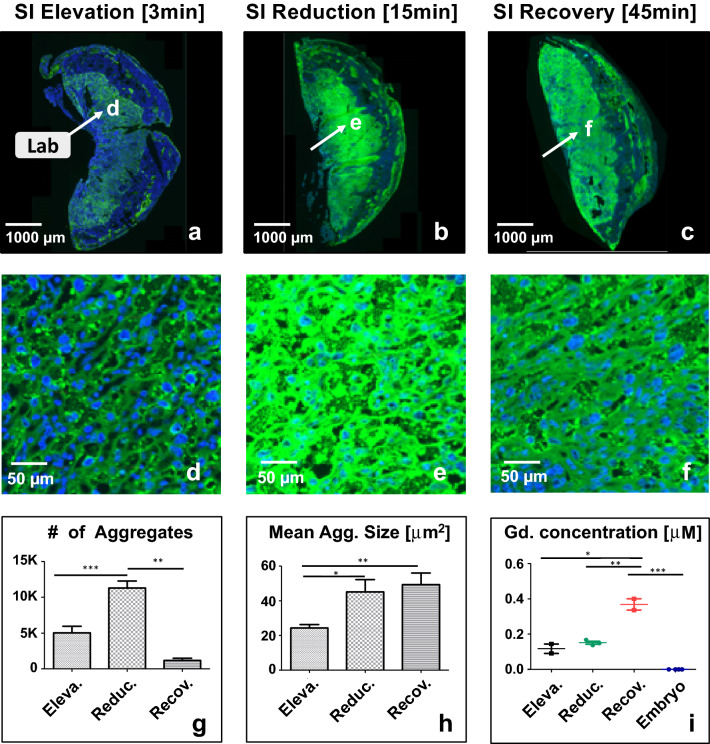
Biotin-transporter mediated internalization of b-BSA-GdDTPA instigates formation of aggregates within labyrinth complex. (**a**–**f**) Fluorescent images of placental histological sections harvested from E14.5 pregnant mice at different time points following tail-vein administration of b-BSA-GdDTPA: (**a**,**d**) 3.5 min, (**b**,**e**) 15 min and (**c**,**f**) 45 min, representing signal elevation (Eleva.), reduction (Reduc.), and recovery (Recov.) phases respectively. Aggregates were automatically labeled by a Fiji-based algorithm and segmented as a yellow mask. Blue, nuclear staining with DAPI. Green, contrast agent (b-BSA-GdDTPA) labeling with Avidin-FITC. (**g**–**i**): Quantitative analysis of aggregate morphology corresponding to the three kinetic phases. (**g**) Total number of aggregates. (**h**) Mean aggregate size. (**i**) ICPMS analysis of Gd concentration in placentas and embryos harvested from each kinetic phase. Mean ± S.E.M. are shown, ** = *p* < 0.01; *** = *p* < 0.001 (*p* value based on t test).

Beyond its direct effect as a T_1_-shortening agent, b-BSA-GdDTPA affected relaxation rates via the formation of aggregates (Fig. 3). These influence the MRI signal in a manner that does not follow the pattern associated with soluble Gadolinium (Eq. 5), but rather create strong microscopic distortions of the local magnetic field, which lead to significant changes in *R*_2_^*^ and attenuate the signal up to two orders of magnitude higher than those caused by soluble gadolinium. Mathematically, this effect was incorporated into Eq. (5) by adding an aggregate attenuation term,$R_{2,Agg}^{*}$,

6 $$S_{spoiled - GRE} (t) = \frac{{M_{0} \sin (\theta ) \cdot \left( {1 - e^{{TR \cdot R_{1} (t)}} } \right)}}{{1 - \cos (\theta )e^{{TR \cdot R_{1} (t)}} }}e^{{ - TE \cdot R_{2}^{*} }} \cdot e^{{ - TE \cdot R_{2,Agg}^{*} }}$$

Due to their large effect on the signal, aggregate formation and clearance were easily identified by analyzing the MRI time-series and marking the time points of signal reduction (aggregate formation) and recovery (aggregate clearance) at each voxel. The output of this procedure were two parametric maps, *T*_formation_ and *T*_clearance,_ representing the transition time-points between the three aggregation phases (signal elevation, attenuation, and recovery).

### Generation of quantitative MRI parametric maps

The time-dependent signal, *S* (*n*) |_n=1…20_, from each voxel in the placenta, was fitted to the theoretical model in Eq. (6). Six unknown parameters exist in the model: the unidirectional exchange rate constants *k*_12_ and *k*_23_*,* the aggregate-induced relaxation rates ($R_{2,Agg}^{*}$) during the elevation, reduction, and recovery phases, and *V*_23,_ the volumetric fraction between the maternal (C2) and fetal (C3) compartments in each voxel. The remaining variables were extracted from the experiment, and included the signal intensity, repetition time, *TR*, echo time, *TE*, and flip angle, *θ*.

To determine the unknown parameters in Eq. (6), we first simulated the signal produced by our MRI protocol on a standard desktop PC. Simulations were implemented using MATLAB (The Mathworks Inc., Natic, MA) and were repeated for a range of physiological parameter values, producing a dictionary of ~ 170,000 different signal time-courses, each corresponding to a unique six-fold set of parameter values [*k*_12_, *k*_23_, $R_{2,Agg}^{*}$@*SI*_Elevation_, $R_{2,Agg}^{*}$@*SI*_Drop_, *V*_23_]]. To determine the values of these parameter at each voxel, we matched the experimental time-curve at each voxel, to the entire set of dictionary entries using exhaustive search. The optimal value was then chosen based on a similarity criterion, defined as the minimal L2-norm between the experimental and simulated curves. The end-product of this process was a set of six parametric maps, describing the exchange rates, *k*_12_ and *k*_23_; contrast-agent aggregation at elevation, reduction, and recovery; and volumetric fraction, *V*_23_, for each imaged slice and for each placenta.

### Histological validation of b-BSA-GdDTPA aggregation and dispersion patterns

MR imaging results were validated by harvesting placentae from 21 pregnant mice at three different time points following contrast administration, corresponding to the three different phases of contrast-agent dynamics during the DCE-MRI scan: (1) initial SI elevation following contrast administration (n = 8, 5 min post administration, Fig. 3a,d); (2) SI reduction (n = 8, 15 min post administration, Fig. 3b,e); and (3) SI recovery phase (n = 5, 45 min post administration, Fig. 3c,f).

Placentae were histologically labeled for b-BSA-GdDTPA, with Avidin-FITC, and analyzed using Fiji image processing and analysis software^13,14^. Fiji procedures were developed and optimized to individually segment aggregates, trophoblast cells, and placentae. A Fiji macro was developed for quantifying aggregation of Avidin-FITC-labeled b-BSA-GdDTPA by segmenting the aggregated structures, excluding the nuclei. This macro segmented distinct aggregates using connected component analysis^15^ and produced the total number of aggregates, their individual areas, and total aggregate area. All values were further normalized by the placenta area. Aggregates were sorted according to size (small < 50 µm, 50 µm < medium < 500 µm, or large > 500 µm) and calculated the number and total area covered by each size group.

To identify trophoblast cells, nuclei were segmented from the blue (DAPI) channel in several steps: (1) Nuclei were enhanced using a bandpass filter, which then were processed with automatic “RenyiEntropy” thresholding method^16^; (2) Small holes in the nuclei were filled and neighboring nuclei were separated using a binary watershed method^17^; (3) Aggregates in the GFP channel were masked by automatic thresholding with the “MaxEntropy” method^18^. Nuclei were excluded from this mask, which was also used for the segmentation of extra-cellular aggregates. Small aggregates (< 5 µ^2^) were ignored. Segmentation of the whole placenta was achieved by combining the GFP and the blue channels into an 8-bit image, and applying a Gaussian blur filter^19^, together with automatic thresholding [“Mean” method], and closing holes using morphological dilation and erosion operations.

### Statistical analysis of aggregate size and count

The relative aggregate area was calculated by dividing total aggregate area by placental area. Groups were compared by one-way ANOVA, followed by a Tukey post-hoc test. Aggregates were also separated into small, medium, and large size groups as defined above, and the same comparison was performed for each size group. The relative number of aggregates was calculated by dividing the number of aggregates by the placenta size. Groups were compared by one-way ANOVA, followed by a Tukey post-hoc test. Comparisons were also made for each size group, as was done for aggregate area.

### Validating the role of biotin transporters using BSA-ROX experiments

To further confirm the involvement of biotin-transporters in the perfusion and aggregation mechanism fluorescent BSA labeled with rhodamine (BSA-ROX, 4 mg in 0.6 ml PBS, Rhenium, Israel) was administered to pregnant B6 mice at E14.5 (n = 4, 12 embryos) via tail vein 60 min prior to animal euthanizing as reported previously^20^. Placentae were fixed, sectioned, and analyzed serially. Histological sectioning was done for only a few consecutive slices, located at the mid sagittal section of the placenta. Placentas were fixed in Carnoy mixture, embedded in paraffin, and sectioned serially at 4-μm thickness.

### Validation of biotin-transporter mediated kinetics using PET imaging

Biotin transporter-mediated kinetics were also validated using PET imaging. Competition experiments were conducted, similar to the MRI study design. In this case [^18^F] Biotin tracer was injected to two groups of pregnant CD1 mice (E18-20): the main experimental (n = 37 fetuses and placentae), and a competition group (n = 39), which was pre-injected with a non-radiolabeled D-biotin in order to test the hypothesis that the accumulation of radiolabeled CA is mediated by biotin transporters. Dynamic PET data were acquired for 20 min, followed by a 5-min CT scan, and concluding with a 40-min dynamic PET scan. Immediately following the 2^nd^ PET acquisitions, mice were sacrificed for biodistribution. Organs and tissues of interest, including each fetus, associated placenta and uterus, were harvested, weighed and the radioactivity was measured using a gamma counter. The uptake, %ID/g, of [^18^F]Biotin in the fetuses and placentae in each group were compared by one-way ANOVA followed by a Tukey’s post-hoc test.

## Results

### Biotin–fluorescein histology reveals biotin transport from the mother to fetus

**Figure 4:**
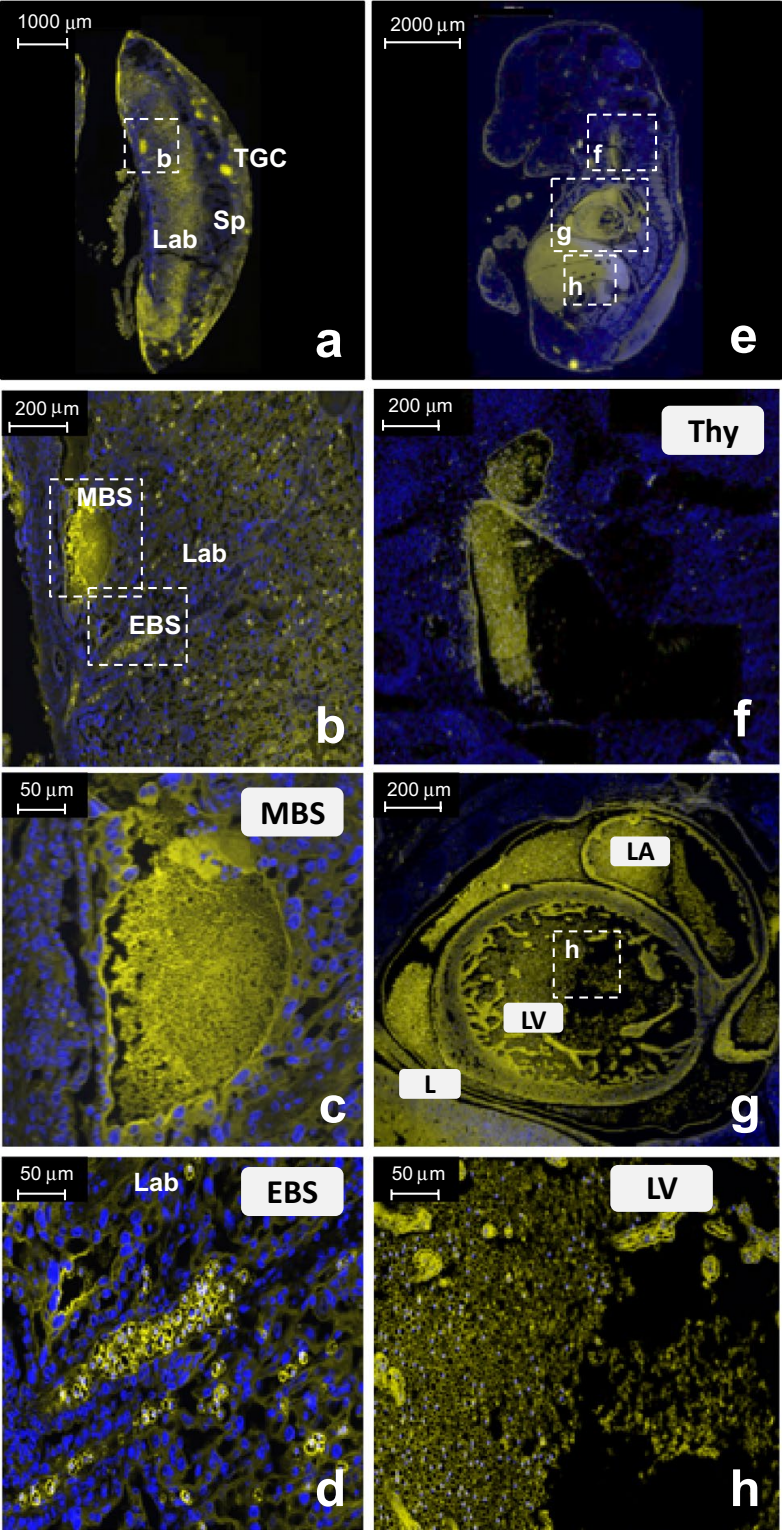
Biotin–Fluorescein injection reveals biotin transport from the mother to the fetus. Fluorescent images of placental and fetal histological sections, harvested from B6 E14.5 pregnant females 60 min post-injection of biotin–Fluorescein via tail vein (**a**–**d**). (**b**) Biotin–Fluorescein was accumulated in the placenta within RBCs in the MBS and fetal RBCs in the EBS of the labyrinth. Higher magnification images are shown for (**c**) MBS and (**d**) EBS. (**e**–**h**) biotin–Fluorescein was transported to the Fetus and accumulated within: RBCs in fetal thymus, heart and liver. Higher magnification images of biotin accumulation within (**f**) the fetal thymus and (**g**) the fetal Left atrium and left ventricle. (**h**) Representative high magnification (50 µm) image of biotin accumulation within the fetal RBCs. Scale bars = 1000 μm (**a**,**e**), 200 μm (**b**,**f**,**g**) and 50 μm (**c**,**d**,**h**). *MBS* maternal blood spaces, *EBS* embryonic blood spaces, *Lab* Labyrinth, *TGC* trophoblast giant cells, *Sp* Spongiothrophoblast cells, *LV* left vertical, *LA* left atrium, *Thy* thymus. Blue, DAPI. Yellow, biotin–fluorescein.

Fluorescent images of biotin within the placentae and adjacent fetuses are shown in Fig. 4, confirming the transport of free biotin from the mother to the fetuses. Following injection of contrast, Biotin–Fluorescein accumulated within red blood cell (RBCs) in the blood spaces of the maternal placenta (MBS) and in the RBCs of the embryonic blood spaces (EBS) in the labyrinth (Fig. 4a–d). In the Fetus, Biotin–Fluorescein accumulated in the fetal heart, liver and thymus (Fig. 4e–h).

### DCE-MRI reveals biotin transporter activity in the placenta

DCE-MRI experiments revealed three unique, complex stages of signal dynamics following the administration of b-BSA-GdDTPA (see Fig. 1b): an initial elevation (0–15 min), a period of signal attenuation (15–30 min), and a signal recovery stage (30–60 min). As validated by histology, signal attenuation was caused by aggregation of the CA. In the competition experiment (where excess native biotin was injected *prior* to administration of b-BSA-GdDTPA), lower initial elevation was observed, signal reduction was eliminated, and the period of signal plateau was extended (Fig. 1b,d).

Signal changes were simultaneous within all the placentae of a litter, and exhibited consistent spatiotemporal directionality: signal attenuation started from the decidua zone, propagated toward the spongiotrophoblast junctional zone (Sp) and ended at the labyrinth zone, without crossing to the fetus due to the contrast agent large size (Fig. 1c). Signal recovery propagated in the opposite direction, i.e., from the labyrinth zone to the decidua, eventually leading to full recovery of placental signal. Signal in the maternal blood vessels (MBV) remained constantly high throughout the three signal phases (Fig. 1b). Additional videos and 3D rendering of the placenta perfusion, with and without biotin administration, are available in the supplementary materials (Fig. S1).

### Histological validation of b-BSA-GdDTPA aggregation and dispersion patterns

Histological analysis of placentae at each kinetic stage revealed aggregates forming in the maternal sinuses and labyrinth, as well as around and within the trophoblast cell layer during the signal elevation stage, right after injection of CA (Fig. 3a,d,g). Aggregate number increased during the signal reduction phase (*p* < 0.001, Fig. 3b,e,g), and then dispersed during the recovery phase (p < 0.01, Fig. 3c,f,g). Aggregate size increased from elevation to reduction phases (*p* < 0.01), with only minor changes observed during the subsequent recovery phase (*p* > 0.05, Fig. 3h). The total area and perimeter of aggregates were congruent with the signal elevation-attenuation-recovery pattern (*p* < 0.001, Fig. S2). Analysis of aggregate distribution revealed maximal area coverage during the SI reduction phase (6.72%) compared with the elevation (1.73%) and recovery (2.1%) phases (*p* < 0.01, Fig. S2a). This suggests a change in aggregate morphology between the three phases. Further analysis of aggregates size-distribution and internal morphology (area and perimeter), revealed three size groups: ≤ 50 μm, 50…500 μm, and ≥ 500 μm, where the majority of aggregates during all phases was smaller than 50 µm (*p* < 0.001, Fig. S2c).

Figure 3i shows the total placental Gd content estimated using ICPMS analysis, demonstrating a gradual accumulation of Gd in the placenta throughout 60 min following administration of b-BSA-GdDTPA. Placental gadolinium content did not vary significantly between the signal elevation and reduction phases, while there was a subsequent × 3.1-fold increase in Gd content during recovery phase (*p* < 0.01). Importantly, Gd content in the fetuses remained undetectable 60 min following contrast administration (*p* < 0.001).

### Biotin competition experiment: histological validation of biotin-transporter-mediated kinetics

**Figure 5:**
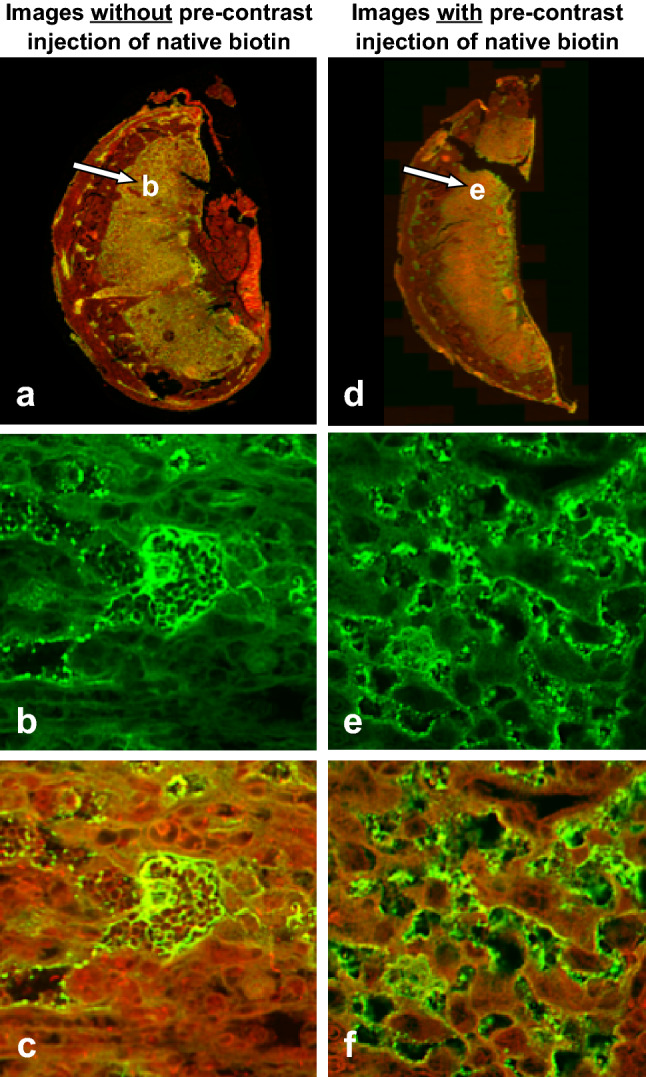
Fluorescence microscopy of placenta from (left) pre-contrast biotin competition experiment, and (right) contrast-only experiments. Histological sections were harvested from a E14.5 pregnant mouse during SI reduction phase [15–30 min]. (**a**–**c**) Representative histological images following the administration of b-BSA-GdDTPA [10 mg, n = 6] for the original experiment, and (**d**–**f**) for biotin-competition experiment involving administration of native biotin (0.2 mg) three min prior to administration of b-BSA-GdDTPA [10 mg, n = 4]. (**b**,**e**) Biotin-only labeling exhibited aggregation of biotin in both versions of the experiment with smaller aggregates occurring for native biotin. (**c**,**f**) Co-labeling for biotin and albumin. The lack of co-localization in (**f**) suggests that aggregates in this panel were formed by native biotin, which preceded to “occupy” the biotin transporters in the placenta. [Green, biotin labeling with Avidin-FITC; Red, Avidin staining for albumin (b-BSA-GdDTPA)].

Biotin competition experiments consisted of injection of excess native biotin prior to injection of b-BSA-GdDTPA. Fluorescent images demonstrate excessive spherical aggregation that did not disperse after 60 min (Fig. 5d–f). The majority of these aggregates were of native biotin, while co-labeling for albumin demonstrated little overlap between biotin and b-BSA-GdDTPA, suggesting the *inhibition of b-BSA-GdDTPA transport and aggregation due to the saturation of the biotin transporters by excess native biotin* (Fig. 5f). On the other hand, stained tissue harvested from the mice injected with only b-BSA-GdDTPA demonstrated almost complete overlap of biotin and albumin staining, lining the perimeter of the maternal sinuses in the labyrinth, ruling out the dissociation of biotin from b-BSA-GdDTPA (Fig. 5a–c).

### Modeling biotin kinetics using a mathematical three-compartment model

**Figure 6:**
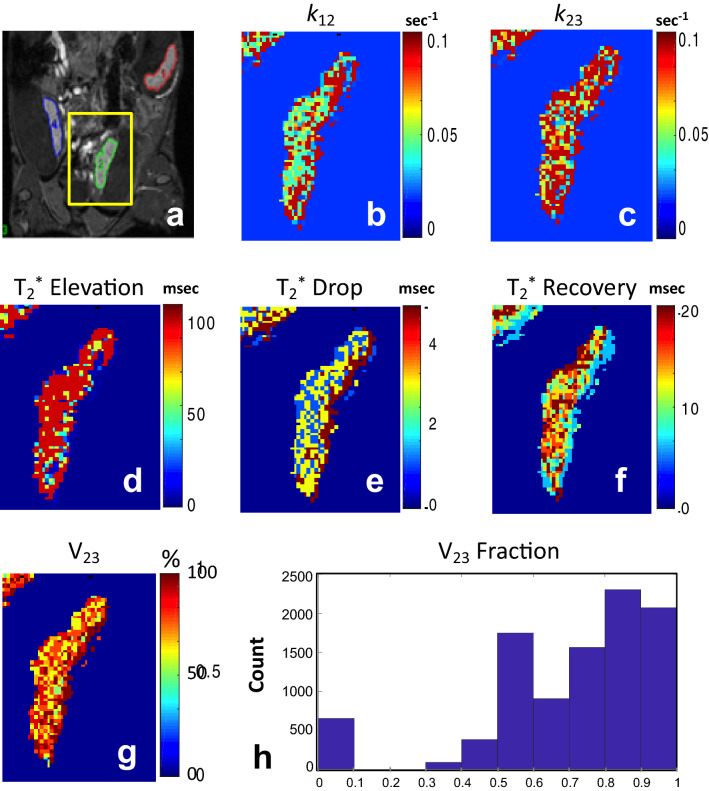
Resolving biotin mediated aggregates assembly and dispersion kinetics based on a three-compartment model. (**a**) T1-weighted 3D-GRE image of a representative placenta. Voxel-by-voxel analysis was performed on all placentae producing six parametric maps: (**b**) *k*_12_ map of the contrast material unidirectional exchange rate from the maternal vasculature (C1) to the placental intravascular compartment (C2). (**c**) *k*_23_ map of the exchange rate between C2 to trophoblast cells compartment (C3). (**d**–**f**) $T_{2}^{*}$ maps of the placenta during the three kinetic phases: elevation, reduction, and recovery. $T_{2}^{*}$ values changed in tandem with the formation and clearance of contrast agent aggregation pattern (see Eq. 6). (**g**) V_23_ map, representing the percentage of C2 and C3 compartments in a chosen slice. (**h**) Histogram of V_23_ values for the placenta.

Figure 6 presents parametric maps for a representative placenta, calculated based on the three-compartment model (Methods "Three-compartmental model of placental architecture" section). The model consists of C1: maternal arterial input, C2: maternal intravascular compartment, and C3: the layer of trophoblast cells (see Fig. 2). Fitting the experimental data to the model produced six parametric maps with the following average values (collected across all mice): inter-compartmental unidirectional exchange rate constants *k*_12_ = 0.045 ± 0.034 s^−1^, and *k*_23_ = 0.059 ± 0.035 s^−1^; three relaxation time constants linked to contrast-agent aggregates during the three signal phases: $T_{2,Elevation}^{*}$ = 63.3 ± 39.7 ms, $T_{2, Reduction}^{*}$ = 2.7 ± 1.6 ms, and $T_{2, Recovery}^{*}$ = 13.6 ± 10.0 ms. The last map shows the average volume fraction *V*_23_ between the maternal blood pool C2, and trophoblast compartment C3. A *V*_23_ value of 0.75 ± 0.25 was produced by the model, signifying that an average of 75% of the pixels belonged to C2 and 25% to C3 (Fig. 6g). A histogram of *V*_23_ values is displayed in Fig. 6h. Representative fitting of simulated to experimental signal curves are illustrated in supplementary Fig. S3.

### Validating the role of biotin transporters using BSA-ROX experiments

No aggregation was observed in the placentas after injection of only BSA, unlabeled by biotin. This suggests that the aggregation of contrast-agent is indeed mediated by a cellular biotin-transporter mechanism (see Fig. S5).

### Validation of biotin-transporter mediated kinetics via fluorine-18 radiolabeled biotin PET imaging

**Figure 7:**
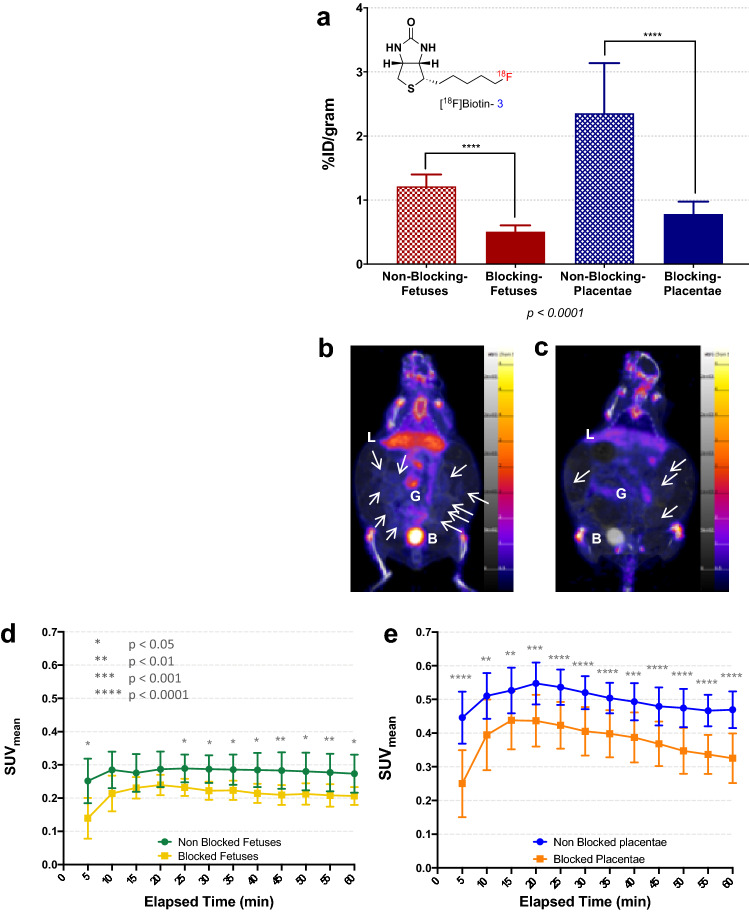
(**a**) Comparative biodistribution of [^18^F]Biotin (structure shown) of fetuses and placentae under non-blocking and blocking conditions. Biodistribution data was obtained ~ 1 h post injection/post PET. (**b**) A summed coronal slice view (55–60 min) of an entire mouse under non-blocking conditions. White arrows are pointing to visible placentae. L = liver, G = gut, B = bladder. (**c**) A summed coronal slice view (55–60 min) of an entire mouse under blocking conditions. White arrows are pointing to visible placentae. (**d**) Average standard uptake values over time in the fetuses of the same non-blocking mouse (**b**, n = 15) and blocking mouse (**c**, n = 10). (**e**) Average standard uptake values over time in the placentae of the same mice. Standard uptake values were calculated from the regions of interests drawn around the fetuses and placentae.

Figure 7a shows the structure of the biotin derivative studied (Fig. S6) as well as the relative uptake in the fetuses and placentae from three dams under non-blocking and three dams under blocking conditions. Fetuses from each litter were grouped together producing an average uptake in the non-blocking fetuses (n = 39) and placentae of 1.3 ± 0.2%ID/g and 2.8 ± 0.8%ID/g, respectively. Uptake of the tracer under blocking conditions (whereupon 1 mg of non-radiolabeled D-biotin was co-injected with the tracer) averaged at 0.47 ± 0.08%ID/g and 0.7 ± 0.2%ID/g in the fetuses and placentae (n = 37/tissue of interest). The difference in uptake between each set of organs was statistically significant.

Figure 7b,c shows the biodistribution of the radiolabeled biotin derivative in a mouse under normal conditions and under blocking conditions during the last 5 min of the 2nd PET acquisition. Both images are windowed to the same level. Figure 7d,e present averaged time activity curves (TAC) of the uptake of the tracer over time in the fetuses and placentae of the mice shown in Fig. 7b,c. Data are presented as the summed standard uptake value [SUV_sum_] versus time [min]. Uptake of the tracer was higher under non-blocking conditions *vs.* under blocking conditions with the co-injected biotin.

## Discussion

### Biotin transporter-mediated uptake and retention of biotinylated contrast agent

This study introduces MRI-based method for in vivo monitoring of biotin transporter kinetics in the murine placenta. We previously reported that administration of b-BSA-GdDTPA resulted in its active uptake by trophoblast cells in the labyrinth and by TGC cells, although the mechanism underlying this uptake remained unclear^21,22^. This study shows that biotin-BSA-GdDTPA uptake is mediated by biotin transporters residing on the trophoblast cells. Following initial MRI signal elevation due to perfusion of the biotinylated CA to the maternal placental blood pool, signal attenuation occurred due to aggregation of the contrast-agent in the maternal blood sinuses, the labyrinth, and their interface with the trophoblast cells, but not in the maternal spiral arteries. Later, MRI signal recovered, as aggregates underwent conformational change and recycling, indicative of an intracellular cell-mediated recycling mechanism. Our results suggest that the formation of aggregates is one of the biological mechanisms, used to regulate the transport of biotin to the fetus. The trophoblast cells use this mechanism to control retention and clearance of biotin, thereby possibly protecting the fetus from undesired fluctuations in nutrient transport and maintaining steady delivery of biotin.

To confirm the involvement of biotin in this recycling mechanism, we performed several validation experiments. First, no aggregation was observed when equal concentration of fluorescent albumin (BSA-ROX) was administered to E14.5 mice. Second, excess native biotin was injected *prior* to the administration of the b-BSA-GdDTPA. This resulted in primarily pure biotin aggregates, as opposed to aggregation of b-BSA-GdDTPA. The injection of native biotin also eliminated the MR signal attenuation, by preventing the aggregation of b-BSA-GdDTPA. This confirms that native biotin and b-BSA-GdDTPA compete for the same trophoblast cell surface biotin transporters.

The specific contrast agent design used in this study affected trans-placental kinetics mainly due to the conjugation of biotin with the large Albumin molecule. This design enabled to isolate the activity of the transporter on the trophoblast surface while preventing its subsequent transfer into the fetal blood stream. The addition of the Gadolinium agent had a low effect on the dynamics, as it does not significantly increase the complex size. Complementing this picture were the fluorescence experiments, showing that low molecular weight biotin-only conjugates did cross into the fetal bloodstream utilizing the same transporter.

### Twofold ${\varvec{T}}_{2}^{{*}}$ effect: soluble gadolinium and aggregates of contrast agent

Contrast-agent aggregates formed when more than 10 mg/kg of b-BSA-GdDTPA was injected (supplementary Fig. S4). Gd can contribute to changes in $T_{2}^{*}$ via two different mechanisms. The first is field heterogeneity around the soluble gadolinium chelate, operating at a molecular (nanometer) length scale. This effect is governed by diffusion of water molecules around the Gd complex, which is being randomly “sampled”, thereby shortening $T_{2}^{*}$ and increasing signal attenuation. The second mechanism is biotin-transporter-mediated formation of CA aggregates, operating at a cellular (micrometer) length scale. The mechanism was denoted as $T_{2,Agg}^{*}$, and was up to two orders of magnitude stronger vis-à-vis soluble CA. Due to this sizable effect, it was the CA aggregates which dominated the MRI signal drop and recovery, easily identified on the DCE-MRI time-series. The spatial distribution of $T_{2,Agg}^{*}$ values reflected two distinct populations, attributed to two functional compartments (C2 and C3). Globally, aggregation-related signal drop exhibited spatial directionality starting at the decidua and the distal labyrinth, and propagating towards the labyrinth. Later, aggregate dispersion and signal recovery propagated back from the labyrinth toward the peripheral labyrinth and venous backflow through the junctional zone and decidua. These patterns correlated well with histology, showing a clear conformational change between signal drop and recovery stages, and supporting the dominant aggregate-mediated $T_{2}^{*}$ effect in the maternal compartment, and the minor $T_{2}^{*}$ effect within the intracellular trophoblast cells.

### Three compartment model

MRI-based analysis of placenta perfusion is used frequently in animal studies and may have clinical utility in predicting pregnancy outcome^15^. While most studies report averaged perfusion values across the entire placenta, recent works suggest that perfusion differs significantly across different regions of the placenta^6,15^. Another factor, which confounds the dynamics of contrast enhancement and should be considered, are specific molecular recognition and transport. Specifically, this study demonstrated the importance of accounting for both placental perfusion and transporter mediated uptake.

The three-compartment model presented herein includes a maternal arterial input (C1), and two placental compartments: an inert maternal, intravascular compartment (C2), and a trophoblast-cell, intracellular compartment in which biotin is actively transported, accumulated and processed (C3). The three-compartment analysis of the MRI signal was based on the histologically validated premise that each voxel in the macroscopic MRI image, is composed of a heterogeneous mixture of compartments C2 and C3, whereas C1 can be easily identified and segmented as a spatially separate compartment. This architecture poses a considerable challenge for in vivo investigations, constraining most studies to the use of ex vivo methods, such as histology and corrosion casts^3^. Here, we incorporated the intricate placental architecture into a comprehensive spatiotemporal model of DCE-MRI signal. Contrast enhancement was excluded from the fetus by using a high molecular-weight b-BSA-GdDTPA that does not cross the placental barrier. The analysis of placental perfusion and biotin transport was thus not confounded by transfer of b-BSA-GdDTPA to the fetal circulation.

The significant correlation between the MRI model predictions and histology validates the functional separation of the labyrinth region of the placenta into two distinct compartments, C2 and C3, thus confirming the three-compartment hypothesis. The exact fractional ratio of compartments varied between placentae and mice, with an average of 75% C2 and 25% C3. Our *k*_12_ and *k*_23_ maps denote the inter-compartmental exchange rates, and reflect characteristic placental physiology, in which the fast exchange (C3) and slow exchange (C2) compartments are meshed in a heterogeneous manner. These findings provide new insights into placenta microstructure, molecular recognition, and transport machinery, adding to previous studies that focused on placental perfusion while assuming a clear spatial separation between the two compartments^6,15^. The quantitative model includes several parameters of interest: the exchange rates reflect whether biotin perfuses normally into and within the placenta; the extent of CA aggregation reflects the efficiency of the active transport mechanism; and the relative fractions of C2 and C3 manifest the placental cellular composition and whether the spiral arteries and trophoblast cells are normally distributed.

Calculated *k*_12_ and *k*_23_ values were consistent with previous reports on biotin transport^22,23^. Accordingly, slightly lower *k*_12_ exchange rates are associated with the rapid flow of maternal blood into the C2 maternal compartment via large, relatively unobstructed arterial canals (C1 compartment). No aggregation was observed in these endothelial blood vessels, corresponding to the passive transport of fluids and contrast-agent into the C2 placental compartment. Trophoblast cells (C3 compartment) line the lumen of the maternal C2 compartment and mediate filtration of water and nutrients to the fetus. However, *since there is no transport of b-BSA-*GdDTPA *to the embryo, the agent accumulates quickly and aggregated outside the trophoblast cells*, consistent with the slightly higher *k*_23_ exchange values. Lastly, b-BSA-GdDTPA aggregation and dispersion kinetics, suggest an active, biotin-dependent, recycling mechanism after biotin passively reaches the C2 compartment.

### PET based validation of biotin distribution

^18^F uptake in the fetuses and placentae indicated that the uptake of the tracer could be inhibited with a co-injection of the non-radiolabeled D-biotin compound. On average, the summed SUV in each mouse was higher in the non-blocking mouse *vs.* that of the blocking mouse, a conclusion that aligned with the biodistribution data. PET experiments were thus in agreement with the MRI findings, further corroborating the conclusion that biotin transport is actively mediated by biotin transporters.

A more accurate quantitative comparison of kinetic parameters from two modalities is not feasible due to the fact that the physical mechanism behind the contrast agents’ influence on the signal is different for MRI and for PET. Another, not less important factor, is that the high sensitivity of PET could be used for analysis of trace levels of biotin, while MRI requires much higher contrast media concentration (above physiological levels). However, while PET can detect only the *amount* of biotin in the tissue, MRI was sensitive to changes in the localization and intracellular environment of the contrast material, including aggregation and clearance.

## Conclusions

This study reports two key advancements. The first is a demonstration that the transport of biotin to the fetus is actively mediated by placental biotin transporters resulting in biotin retention in intracellular aggregates. Our experiments revealed an active biotin uptake, retention, and clearance mechanism, as well as its spatiotemporal distribution in the placenta. The second advancement is the introduction of a new MRI-based tool that can accurately and reliably probe the structure and perfusion in murine placenta in vivo, as well as the dynamics of biotin transport and intracellular aggregation. The tool is based on a biophysical model of the placenta, which incorporates its microanatomy and physiology as well as the molecular dynamics of biotin transporter activity, into a comprehensive MRI signal model. The presented methodology can assist in monitoring biotin deficiency or impaired biotin transport to the fetus during pregnancy or treatment follow up, and may be relevant also to other essential vitamins^24,25^.

### Supplementary information

Supplementary Information.

Supplementary Video 1.

Supplementary Video 2.

## Data Availability

All data used in this study will be shared upon request.

## Appendix: Three-compartment model—mathematical formalism

This appendix describes the mathematical formalism for the dynamics of contrast agent flowing into the placenta and its transporter mediated uptake and processing. The model is illustrated in Fig. 2 and is based on contrast material first filling C1, the maternal arterial bed, which is much larger relative to C2 and C3, the placental maternal and trophoblast-cell compartments. As a result, C1 acts as a constant-concentration contrast material input to the other two compartments. The constant concentration in C1 was calculated to be approximately 100 mM based on the amount of contrast material injected divided by blood volume. The master equations governing the exchange of contrast material between the three compartments are

7 $$\begin{aligned} \dot{c}_{2} & = k_{12} \left( {c_{1} - c_{2} } \right) - k_{23} \left( {c_{2} - c_{3} } \right) \\ \dot{c}_{3} & = k_{23} \left( {c_{2} - c_{3} } \right) \\ \end{aligned}$$

where *c*_1_, *c*_2_, and *c*_3_, denote the concentrations in the three compartments and *k*_*ij*_ denotes unidirectional exchange between compartment *i* and *j*. The sign of *k*_*ij*_ signifies inflow or outflow. To solve these equations, we first use the second equation to isolate the first and second derivative of c_2_:

8 $$\begin{aligned} c_{2} & = \frac{{\dot{c}_{3} }}{{k_{23} }} + c_{3} \\ \dot{c}_{2} & = \frac{{\ddot{c}_{3} }}{{k_{23} }} + \dot{c}_{3} \\ \end{aligned}$$

Plugging these expressions back into equation Eq. (7) yields,

9 $$\begin{gathered} \ddot{c}_{3} k_{23}^{ - 1} + \dot{c}_{3} = k_{12} \left( {c_{1} - \dot{c}_{3} k_{23}^{ - 1} - c_{3} } \right) - k_{23} \left( {\dot{c}_{3} k_{23}^{ - 1} + {\raise0.7ex\hbox{$c$} \!\mathord{\left/ {\vphantom {c 3}}\right.\kern-\nulldelimiterspace} \!\lower0.7ex\hbox{$3$}} - {\raise0.7ex\hbox{$c$} \!\mathord{\left/ {\vphantom {c 3}}\right.\kern-\nulldelimiterspace} \!\lower0.7ex\hbox{$3$}}} \right) \hfill \\ \ddot{c}_{3} k_{23}^{ - 1} + \dot{c}_{3} = k_{12} c_{1} - k_{12} k_{23}^{ - 1} \dot{c}_{3} - k_{12} c_{3} - \dot{c}_{3} \hfill \\ \ddot{c}_{3} k_{23}^{ - 1} + \dot{c}_{3} \left( {2 + k_{12} k_{23}^{ - 1} } \right) + c_{3} k_{12} - c_{1} k_{12} = 0 \hfill \\ \end{gathered}$$

We next change variables according to

10 $$\begin{array}{*{20}l} {d \triangleq c_{3} k_{12} - c_{1} k_{12} } \hfill & \to \hfill & {c_{3} = d/k_{12} + c_{1} } \hfill & {(a)} \hfill \\ {\dot{d} = \dot{c}_{3} \left( {k_{12} k_{23} } \right)} \hfill & \to \hfill & {\dot{c}_{3} = \dot{d}/k_{12} } \hfill & {(b)} \hfill \\ {\ddot{d} = \ddot{c}_{3} \left( {k_{12} k_{23} } \right)} \hfill & \to \hfill & {\ddot{c}_{3} = \ddot{d}/k_{12} } \hfill & {(c)} \hfill \\ \end{array}$$

This allows to rewrite Eq. (9) as a simplified second order differential equation of the form

11 $$\ddot{d}\left( {k_{12}^{ - 1} k_{23}^{ - 1} } \right) + \dot{d}\left( {2k_{12}^{ - 1} + k_{23}^{ - 1} } \right) + d = 0$$

*d* can be judiciously guessed to have the following form:

12 $$d = Ae^{\beta t}$$

yielding

13 $$Aa_{2} e^{\beta t} \beta^{2} + Aa_{1} e^{\beta t} \beta + Aa_{0} e^{\beta t} = 0,$$

which can only be true if

14 $$a_{2} \beta^{2} + a_{1} \beta + a_{0} = 0.$$

The last expression can be solved using a simple quadratic formula

15 $$\beta_{12} = \frac{{ - a_{1} \pm \sqrt {a_{1}^{2} - 4a_{2} a_{0} } }}{{2a_{2} }} = - k_{23} - 0.5 \cdot k_{12} \pm \sqrt {k_{23}^{2} + 0.25 \cdot k_{12}^{2} }$$

The solutions to c_3_ and c_2_ can now be calculated (in that order) based on Eqs. (10) and (8) producing

16 $$\begin{aligned} c_{2} & = Ae^{{\beta_{1} t}} k_{12}^{ - 1} \left[ {1 + \beta_{1} k_{23}^{ - 1} } \right] + Be^{{\beta_{2} t}} k_{12}^{ - 1} \left[ {1 + \beta_{2} k_{23}^{ - 1} } \right] + c_{1} \\ c_{3} & = \left( {Ae^{{\beta_{1} t}} + Be^{{\beta_{2} t}} } \right)k_{12}^{ - 1} + c_{1} \\ \end{aligned}$$

### Boundary condition at time *t* = 0

At time *t* = 0 the CA concentrations in the placenta is zero. This can be expressed as

17 $$\left\{ {\begin{array}{*{20}c} {c_{2} \left( {t = 0} \right) = 0} \\ {c_{3} \left( {t = 0} \right) = 0} \\ \end{array} } \right.$$

Plugging in the expressions for *c*_2_ and *c*_3_ and rearranging terms, we obtain the solution to the constants *A* and *B*:

18 $$A = c_{1} k_{12} \beta_{2} /\left( {\beta_{1} - \beta_{2} } \right)$$

19 $$B = - c_{1} k_{12} \beta_{1} /\left( {\beta_{1} - \beta_{2} } \right)$$

### Asymptotic behavior at time *t* = ∞

Before examining the asymptotic behavior of the concentrations, we draw attention to the sign of the constants *β*_1_ and *β*_2_. Assuming, without loss of generality, that all exchange rates are nonnegative, it can be shown from (15) that both constants are always negative. It directly follows that the exponential terms in (16) will reduce to zero at later times, leading both c_2_ and c_3_ to be equal to c_1_

20 $$\begin{aligned} c_{2} |_{t = \infty } & = c_{1} \\ c_{3} |_{t = \infty } & = c_{1} \\ \end{aligned}$$

indicating that the system eventually reaches a natural equilibrium across the three compartments C2, C3 and C1.

### Translating concentrations to MR relaxation rates

The time-dependent concentrations within each compartment were used to calculate the corresponding changes in the R_1_ and R_2_ relaxation rates, using typical relaxivity (R_1_) values^12^:

21 $$R_{1}^{(C2,C3)} (t) = c_{2,3} (t)\,[{\text{mM}}] \times 120\,[\sec^{ - 1} \,{\text{mM}}^{ - 1} ]$$

22 $$R_{2}^{(C2,C3)} (t) = c_{2,3} (t)\,[{\text{mM}}] \times 17\,[\sec^{ - 1} \,{\text{mM}}^{ - 1} ]$$

Finally, these values were set into the steady-state signal equation for GRE MRI^12^ yielding the time dependent signal arising from each voxel in the MRI images

23 $$S_{spoiled - GRE} (t) = \frac{{M_{0} \sin (\theta ) \cdot \left( {1 - e^{{TR \cdot R_{1} (t)}} } \right)}}{{1 - \cos (\theta )e^{{TR \cdot R_{1} (t)}} }}e^{{ - TE \cdot R_{2}^{*} }}$$

where $\theta$ denotes the excitation flip angle, *M*_0_ the proton density weighted by the receiver coil sensitivity profile, and TR, TE the repetition and echo time, respectively.

## Abbreviations

b-BSA-GdDTPA: Biotin-bovine serum albumin-gadolinium diethylenetriaminepentaacetate
VC: Vena cava
DCE-MRI: Dynamic contrast enhanced magnetic resonance imaging
3D-GE: Three-dimensional gradient echo
BSA: Bovine serum albumin
ICPMS: Inductively coupled plasma mass spectrometry
SI: Signal intensity
RARE: Rapid imaging with refocused echoes
TGC: Trophoblast giant cells
TS: Trophospongium
%ID/g: Percent injected dose per gram of tissue

## Code availability

Simulations and signal processing code packages will be provided upon request.
