# Author Correction: Novel multimodal molecular imaging of Vitamin H (Biotin) transporter activity in the murine placenta

**DOI:** 10.1038/s41598-021-85422-z

**Published:** 2021-03-15

**Authors:** Noam Ben-Eliezer, Marina Lysenko, Inbal E. Biton, Ofra Golani, Jennifer L. Bartels, Solana R. Fernandez, Tolulope A. Aweda, Nicholas A. Clanton, Rebecca Beacham, Suzanne E. Lapi, Joel R. Garbow, Michal Neeman

**Affiliations:** 1 Department of Biological Regulation, Weizmann Institute of Science, 7610001 Rehovot, Israel; 2 Department of Biomedical Engineering, Tel Aviv University, Tel Aviv, Israel; 3 Department of Veterinary Resources, Weizmann Institute of Science, 76100 Rehovot, Israel; 4 Life Sciences Core Facilities, Weizmann Institute of Science, 76100 Rehovot, Israel; 5 Department of Radiology, University of Alabama at Birmingham, Birmingham, AL USA; 6 Department of Chemistry, University of Texas at San Antonio, San Antonio, TX USA; 7 Biomedical MR Laboratory, Mallinckrodt Institute of Radiology, Washington University in St. Louis, St. Louis, MO USA

Correction to: *Scientific Reports* 10.1038/s41598-020-77704-9, published online 27 November 2020

The original version of this Article contained errors in the spelling of the authors Noam Ben-Eliezer, Marina Lysenko, Inbal E. Biton, Ofra Golani, Jennifer L. Bartels, Solana R. Fernandez, Tolulope A. Aweda, Nicholas A. Clanton, Rebecca Beacham, Suzanne E. Lapi, Joel R. Garbow & Michal Neeman which were incorrectly given as Ben Eliezer Noam, Lysenko Marina, Biton E. Inbal, Golani Ofra, Bartels L. Jennifer, Fernandez R. Solana, Aweda A. Tolulope, Clanton A. Nicholas, Beacham Rebecca, Lapi E. Suzanne, Garbow R. Joel & Neeman Michal.

This error has now been corrected in the PDF and HTML versions of the Article and the Supplementary Information file that accompanies the Article.

