# Supplementary material for: Novel multimodal molecular imaging of Vitamin H (Biotin) transporter activity in the murine placenta
Source: Sci Rep. 2020 Nov 27;10:20767. doi: 10.1038/s41598-020-77704-9 (PMC7695856; doi:10.1038/s41598-020-77704-9)
Supplement: Supplementary file 1 — Supplementary Information. [file 41598_2020_77704_MOESM1_ESM.pdf]

SUPPLEMENTARY INFORMATION

for

Novel multimodal molecular imaging of Vitamin H (Biotin) Transporter activity in the Murine Placenta

Dr. Noam Ben Eliezer, PhD[1,2,†], Dr. Marina Lysenko, PhD[1,†], Dr. Inbal E Biton, PhD [3], Dr. Ofra Golani, PhD [4], Dr. Jennifer L Bartels, PhD[5], Solana R Fernandez [5], Dr. Tolulope A Aweda, PhD[5], Nicholas A Clanton [5,6], Rebecca Beacham [5], Prof. Suzanne E Lapi, PhD [5], Prof. Joel R Garbow, PhD [7], and Prof. Michal Neeman, PhD[1*]

[1] Department of Biological Regulation, Weizmann Institute of Science, Rehovot 76100 Israel
[2] Department of Biomedical Engineering, Tel Aviv University, Tel Aviv, Israel
[3] Department of Veterinary Resources, Weizmann Institute of Science, Rehovot 76100 Israel
[4] Life Sciences Core Facilities, Weizmann Institute of Science, Rehovot 76100 Israel
[5] Department of Radiology, University of Alabama at Birmingham, Birmingham, AL United States
[6] Department of Chemistry, University of Texas at San Antonio, San Antonio, TX, United States
[7] Biomedical MR Laboratory, Mallinckrodt Institute of Radiology, Washington University in St. Louis, St. Louis, MO, United States

[*] Corresponding Author
[†] Authors contributed equally to this work

Corresponding author
Prof. Michal Neeman
Department of Biological Regulation,
Weizmann Institute, Rehovot 76100 Israel
Tel 972-8-9342487
Email michal.neeman@weizmann.ac.il

1 SUPPLEMENTARY MATERIALS AND METHODS

1.1 Contrast preparation and administration

Biotin-BSA-GdDTPA [19] was synthesized from bovine serum albumin (BSA) by conjugation with biotin and GdDTPA (biotin-BSA-GdDTPA; approximately 80 kDa; r_1 relaxivity of 177 $mM^{-1}s^{-1}$ per albumin and 7.55 $mM^{-1}s^{-1}$ per Gd at 4.7 T). During dynamic contrast enhanced (**DCE**) MRI experiments, an intravenous bolus dose of biotin-BSA-GdDTPA (10 mg/mouse (Gd: 92 μmol/kg) in 200 μl of PBS) was injected via a tail-vein catheter. The biotin label enabled the distribution of the contrast material to be visualized in histological sections.

1.2 DCE-MRI Imaging and analysis

MRI experiments were performed on a 9.4 tesla BioSpec Magnet 94/20 USR system (Bruker, Germany) equipped with a gradient-coil system capable of producing pulsed gradients of up to 40 gauss/cm in each of the three directions. A quadrature volume coil, with 72-mm inner diameter and a homogeneous RF field of 100 mm along the axis of the magnetic field, was used as both transmitter and receiver. During MRI scanning, mice were anesthetized with isoflurane (3% for induction, 1–2% for maintenance) in O_2 (1 liter/min) delivered through a nasal mask. Once anesthetized, the animals were secured in a head-holder and were imaged in a supine position. Respiration rate was monitored and maintained at 30–45 breaths/min, while body temperature was maintained at 37 °C *via* circulating warm water.

B6 females at E14.5 were divided into two groups: One group (control, n=10) was injected with biotinylated contrast agent (b-BSA-Gd-DTPA, diluted in PBS, SyMO-Chem, Netherlands) a second group (competition, n=5) was injected with native biotin (0.04 mg per mouse, diluted in 200 μl) three minutes prior to administration of b-BSA-Gd-DTPA. For both groups, bolus injection was *via* a tail vein. To determine the position of the multiple embryos within the uterus, T_2-weighted RARE [20] images were collected using the following parameters: TE = 7 ms; $N_{averages}$=4; FOV = 40x40x40 mm^3;

matrix=256x256x64; acquisition time = 4:58 min. For dynamic post-contrast imaging, 20 repeated T_1-weighted 3D-GRE images were acquired for a total of 54 min starting at the time of contrast agent administration, using the following parameters: pulse flip angle = 15°; TR = 10 ms; TE = 3 ms; $N_{averages}$ = 2; FOV = 40x40x40 mm^3; matrix = 256x256x64; N_{scans} = 20; acquisition time = 2:43 min per scan.

1.3 Post-mortem histology, immunohistochemistry, and fluorescence microscopy

Following MRI imaging, pregnant animals were euthanized with an overdose of pentobarbital, uteri were exposed, and embryos were counted, weighed, and numbered according to their position along the uterine horn.

Placentae were fixed in Carnoy mixture, embedded in paraffin, and sectioned serially at 4-µm thickness. Sections were deparaffinized with a xylene substitute (Safeclear II; Fisher Scientific Company LLC, Kalmazoo, MI) for 5 min; rehydrated sequentially with 100%, 95%, and 70% ethanol and double-distilled water for 5 min each, and then equilibrated in PBS for 5 min. Slides were then incubated with Citrate buffer (PH=6) for one hour in a pressure cooker for antigen retrieval. After nonspecific binding was blocked using a solution of 20% normal horse serum and 0.2% of Triton-X in PBS for 1.5 h at room temperature, sections were incubated in blocking solutions containing 2% normal horse serum and 0.2% Triton-X Anti-Bovine Albumin Antibody (1:100, Bio-Rad, Hercules, CA, USA) in PBS. Later, sections were incubated with a blocking solution containing Avidin-FITC (1:100; Sigma-Aldrich, Inc., St. Louis, MO) and cy3-anti-mouse (1:100) in PBS. Following nuclear staining with fluorescent DAPI (4',6-diamidino-2-phenylindole; 1:20,000 dilution of 10 mg/ml) for two minutes at room temperature, sections were mounted with Aqua-Mount medium (Thermo Scientific), scanned with a 3D Histech Panoramic Midi scanner (3DHISTECH Ltd.), and analyzed with 3D Histech Panoramic Viewer software. Statistical analysis was performed with Matlab

[MathWorks, Natick, MA, USA] and R [The R Project for Statistical Computing, https://www.r-project.org/].

1.4 Validation of biotin-transporter mediated kinetics using PET imaging

Synthesis of radiolabeling precursor: [^{18}O]-enriched water was bombarded with a 20-MeV proton beam from the University of Alabama medical cyclotron facility at 40 µA for ~10 min. The free [^{18}F]F$^-$ was trapped onto a wetted QMA cartridge, and then eluted with a 0.5 mL solution containing 2-3 mg of K_2CO_3 into a vial containing 8-10 mg of kryptofix in 1 mL of anhydrous acetonitrile. The free [^{18}F]F$^-$ solution was dried azeotropically with 3 more additions of 1 mL of anhydrous acetonitrile, under constant He flow and at 110°C.

After drying, compound #2 (5 mg) was dissolved into 300 µL of anhydrous dimethyl sulfoxide, and added to the vial and heated at 135° C for 30 min. After the synthesis, the vial was cooled to room temperature and diluted into 4 mL of water. The crude reaction solution was pushed through and trapped onto a C18 cartridge, first prepared with 3 mL of ethanol and 10 mL of water. The trapped product was washed with another 10 mL of water to remove any unreacted [^{18}F]F$^-$. The final product, [^{18}F]biotin (**3**) was then eluted off of the C18 Sep-Pak with 1 mL of ethanol, concentrated down to dryness and reconstituted into 25 µL of ethanol for dose preparation described below. Radiopurity of the final compound was checked with radio-HPLC and was found to be greater than 98%. Radiosynthesis and purification took approximately 1 hour with average decay-corrected yields of 30 % (546 GBq or 147 mCi).

It should be mentioned that while the synthesis of the radiolabeling precursor, #2 **(**see **Fig. S6)**, was accomplished in one step, the precursor was not stable due to a high reactivity. To ensure good radiolabeling yields, the precursor was therefore synthesized no more than one week before the study, and kept under vacuum until use, to ensure a dry environment.

Small animal PET/CT imaging and biodistribution studies of [^{18}F]Biotin in pregnant mice: Immediately before injection of the contrast agent, the [^{18}F]Biotin dose was prepared as follows: (non-blocking) 3.7 MBq (~ 100 μCi), in 0.5-5 μL depending on time of injection) was diluted into an appropriate amount of 1M HEPES, pH 7.1, to a final volume of 100 μL or (blocking) 3.7 MBq (~ 100 μCi) of [^{18}F]Biotin, 7 μL of 0.1 mg/μL D-biotin in DMSO, q.s. to 100 μL of 1M HEPES, pH 7.1. Pregnant CD1 mice (E18-20) were anesthetized with 2-3% isoflurane in oxygen, maintained at normal body temperature, with respiratory monitoring. Animals were first injected with 3.5 mg/g of body weight Omnipaque, an iodine-based CT contrast agent, followed by the prepared Biotin PET tracer described above, also via tail vein injection.

Animals were placed on the scanner bed and dynamic PET data were acquired for 20 min, followed by a 5-minute CT scan (80kVp), concluding with a 40-minute dynamic PET scan. Immediately following the conclusion of the 2nd PET acquisitions, the mouse was sacrificed immediately for biodistribution. Organs and tissues of interest, including each fetus, associated placenta and uterus, were harvested, weighed and the radioactivity was measured using a gamma counter. Data were decay corrected to time of animal sacrifice and calculated as the percent injected dose per gram of tissue (%ID/g).

CT images were reconstructed using a Modified Feldkamp Algorithm. The PET images were reconstructed using a 3D-OSEM (Ordered Subset Expectation Maximization) algorithm (24 subsets and 3 iterations), with random, attenuation, and decay correction. Final images were processed using Vivoquant.

Statistical analysis of uptake of PET tracer in placentae and fetuses: The uptake, %ID/g, of [^{18}F]Biotin in the fetuses and placentae in each group were compared by one-way ANOVA followed by a Tukey's post-hoc test. There were 39 fetuses and placentae for the non-blocking studies and 37 fetuses and placentae for the blocking studies.

2 SUPPLEMENTARY FIGURES

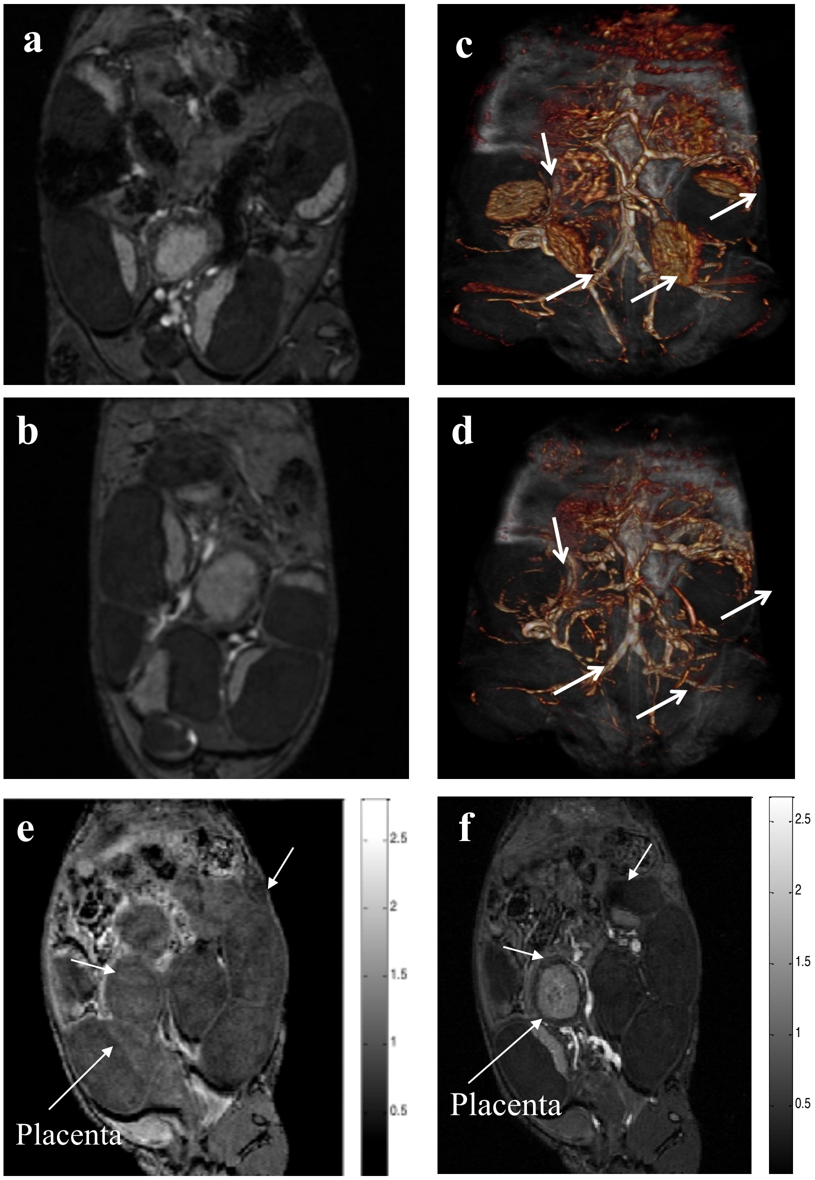

Figure S1: 3D movie and reconstruction, based on T1-weighted images, illustrate SI change through 60 min of DCE-MRI imaging. (a-b) movie of T1-weighted images demonstrating the contrast-agent dynamics of each placenta and the vena cava during 60 min following: **(a)** b-BSA-GdDTPA administration **(b)** native biotin administration three min prior to contrast agent administration. (c-d) Representative 3D reconstruction images of: **(c)** SI enchantment after 5 min and **(d)** SI reduction after 15 min in four different placentas following contrast administration. Placentas are marked with white arrows. **(e)** Pre-contrast T1 weighted images. **(f)** Post-contrast T1 weighted images. Placentas are marked with arrows.

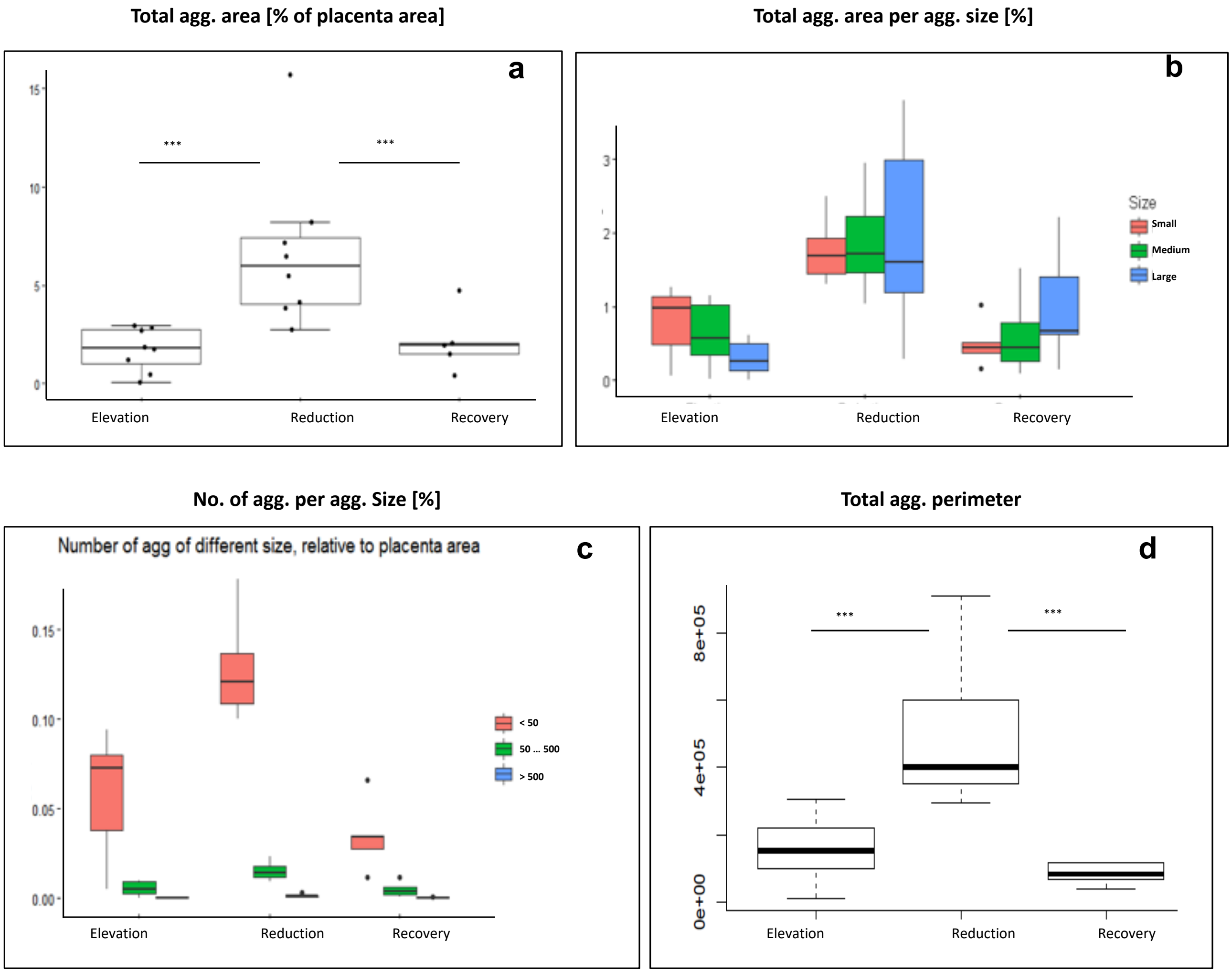

Figure S2: Calculation of segmented aggregates. Quantitative statistical analysis of aggregates corresponding to the three kinetical stages: first SI elevation, SI reduction and SI recovery. **(a)** Total area of aggregates per kinetical stage, as a fraction from placental area [μm^2]. **(b)** Total area of aggregates per kinetical stage, separated into aggregate size [μm^2]. **(c)** Calculation of total number of aggregates per size bin per kinetical stage. **(d)** Total aggregates perimeter per kinetical stage [μm]. All results are normalized to the total placentas area. Mean+ S.E.M. are shown, *=p<0.05, **=p<0.01; ***=p<0.001. p value based on *t* test.

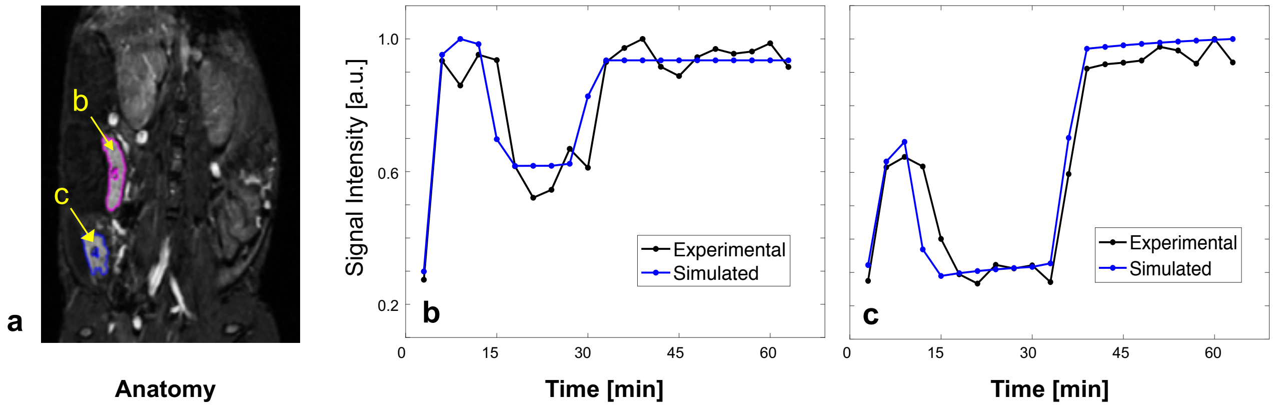

Figure S3: Fitting between experimental (black) signal curves and simulated (blue) signal dynamics based on the suggested three-compartment model. **(a)** Anatomic image of the two samples placentas shown in 'b' and 'c'. **(b-c)** Experimental and simulated signals for two representative voxels, exhibiting three dynamic stages: signal enhancement, drop, and recovery.

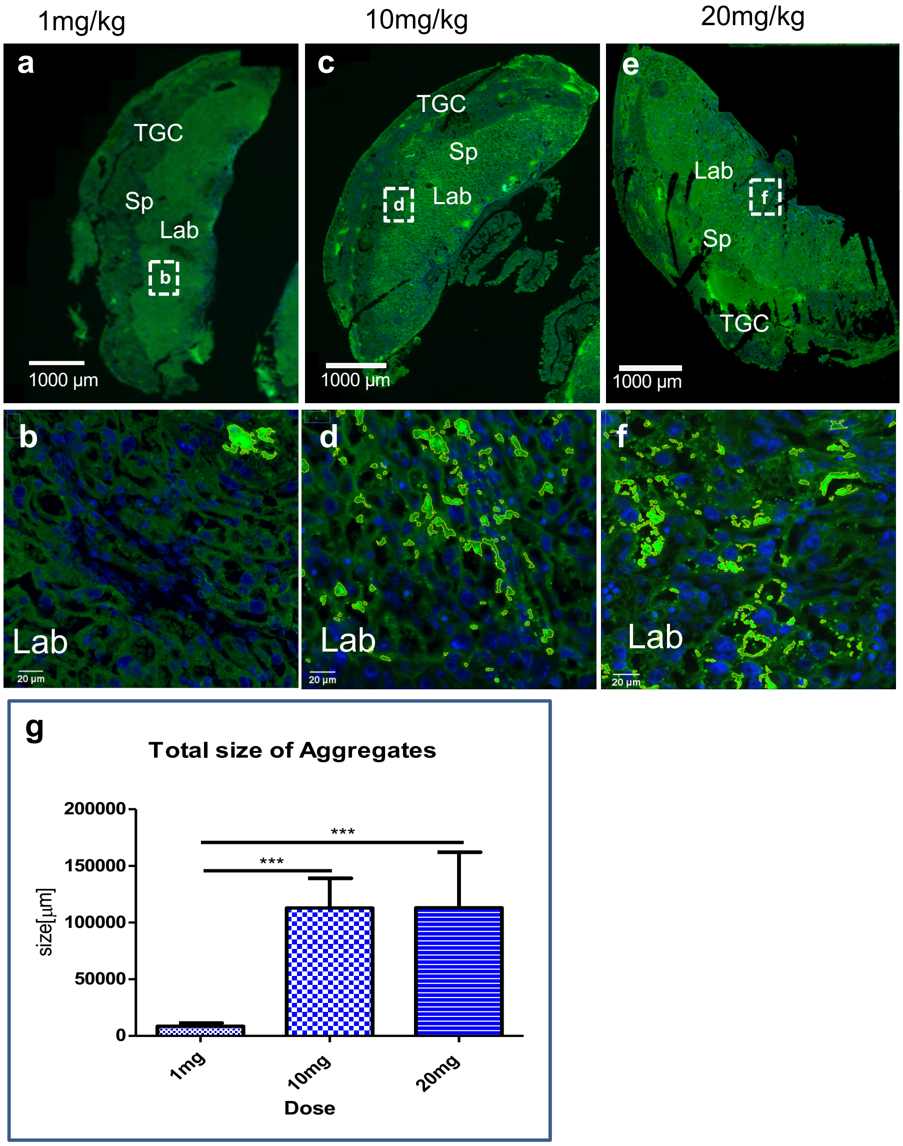

Figure S4: Active uptake and aggregation of the biotinylated contrast agent is observed in high doses. B6 (C57BL/6J) E14.5 pregnant females were injected with three different doses of b-BSA-GdDTPA: 1 mg, 10 mg and 20 mg via the tail vein; Contrast agent (b-BSA-GdDTPA) histological labeling with Avidin-FITC after 60 min of contrast administration demonstrated aggregate formation during (**c-d**) 10mg/kg and (**e-f**) 20mg/kg doses injection; mild aggregate formation was detected at 1mg/kg injection dose (**a-b**). (**g**) Quantitative analysis of total aggregate size at 1mg was 8618 ± 2868 µm (n=9); at 10 mg 113000 ± 26200 µm (n=5); and at 20 mg 113100 ± 48980 µm (n=2). Mean ± S.E.M. are shown. **=p<0.01; ***=p<0.001. p value based on unpaired *t* test.

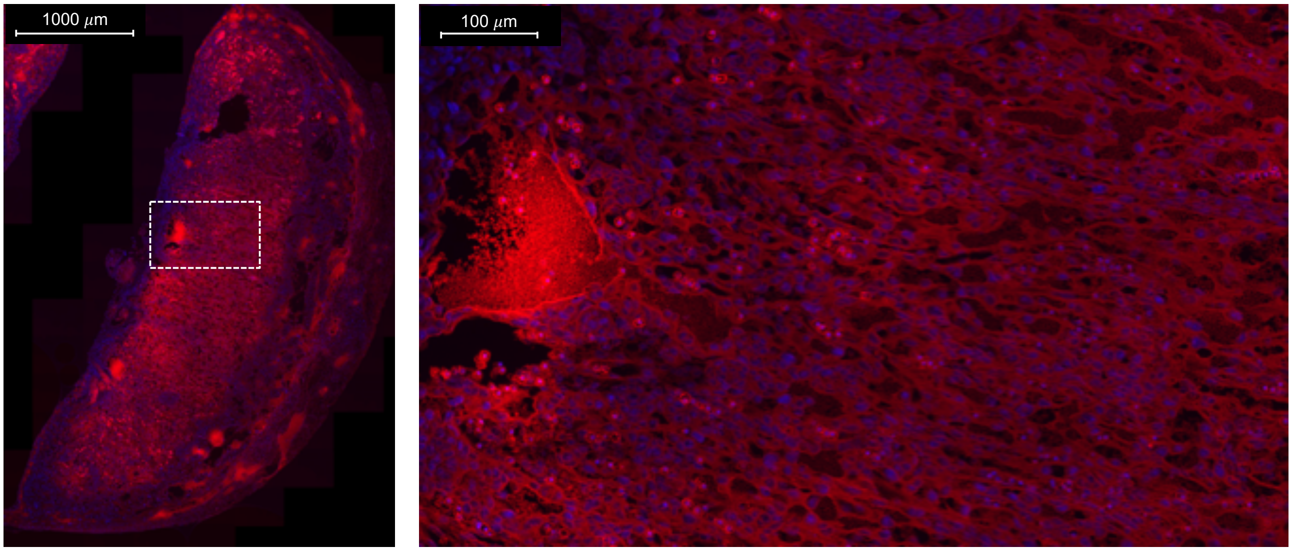

Figure S5: Validation of the involvement of biotin in the mechanism underlying aggregation. Labeled albumin (BSA-ROX) only was administered to E14.5 mice in a similar molar concentration as the biotinylated contrast agent (b-BSA-GdDTPA). No aggregation was observed in the placentas in this case, suggesting that the coupling of biotin is the factor that mediates contrast aggregation through a cellular biotin transporter mechanism.

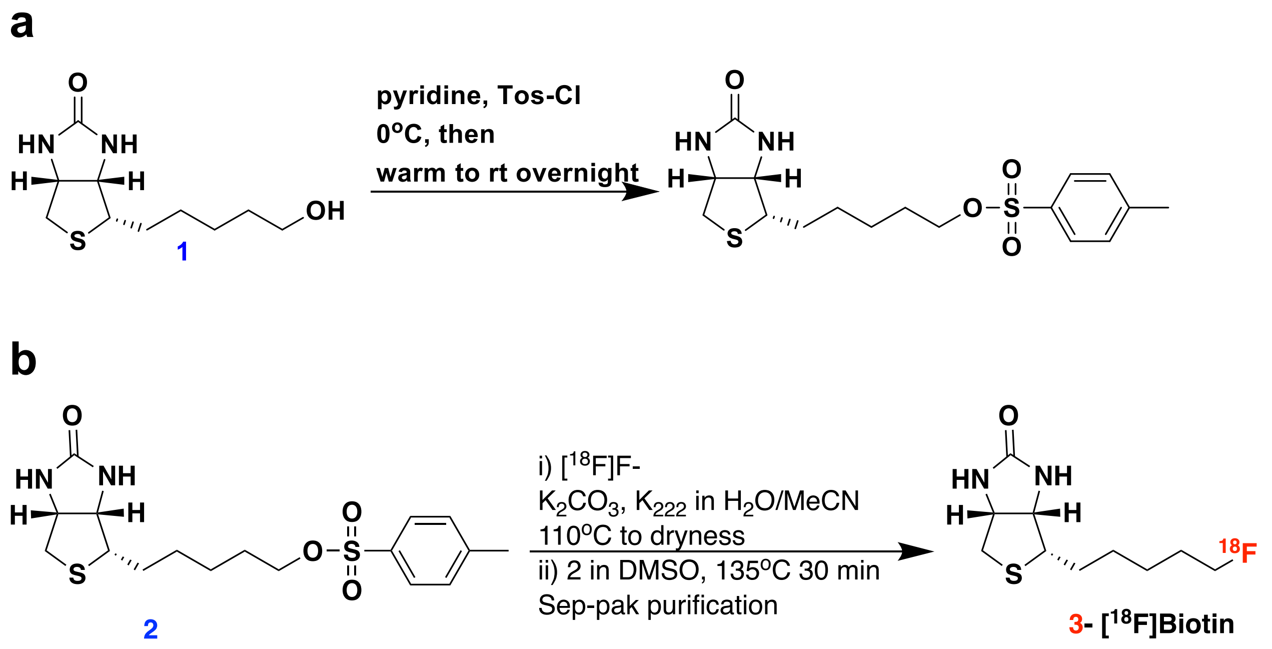

Figure S6. (a) Synthetic route for the preparation of the radiolabeling precursor. Details described below. **(b)** Synthetic scheme for the radiosynthesis of [^{18}F]Biotin. Details described in section 5.16 in the article.

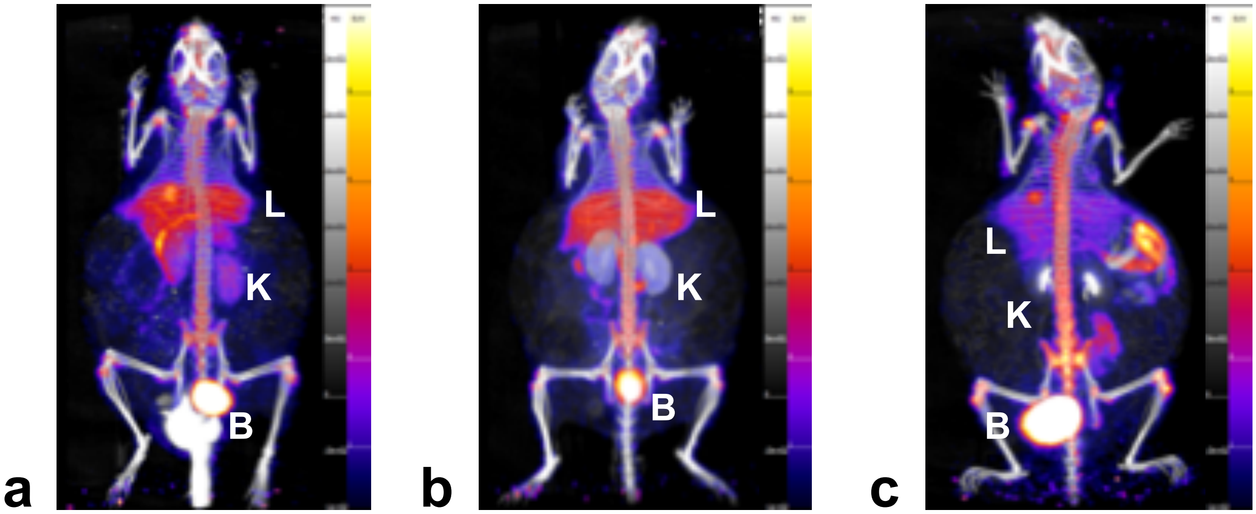

Figure S7. Images a, b, and c above are summed maximal intensity projections (MIPs) of combined PET and CT of 3 mice who received the tracer [^{18}F]Biotin. L = liver, K = kidney, B = bladder

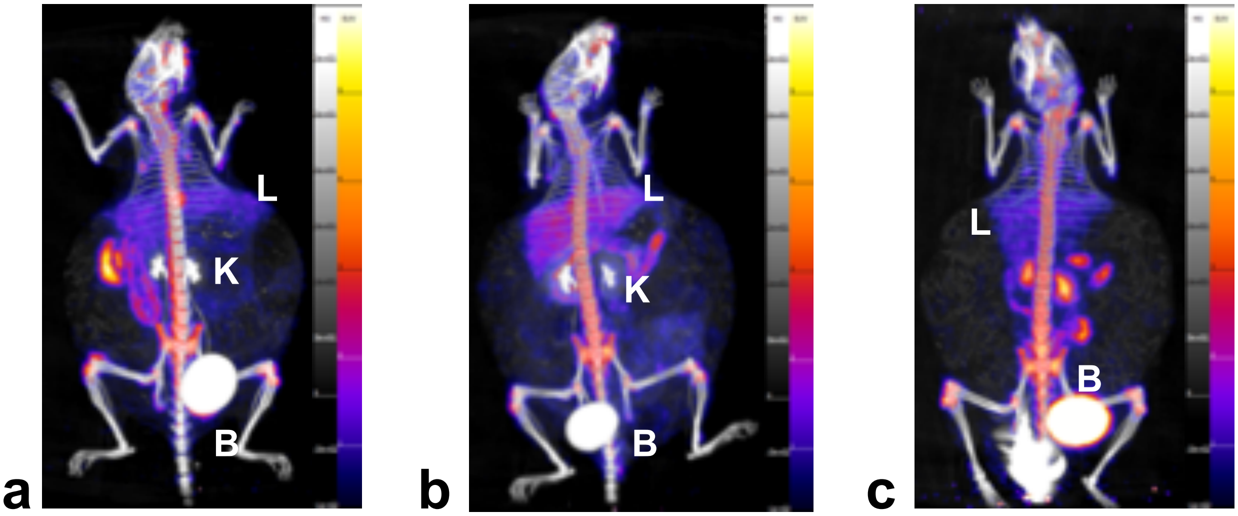

Figure S8. Images a, b, and c above are summed maximal intensity projections (MIPs) of combined PET and CT of 3 mice who received the tracer [^{18}F]Biotin plus a co-injection of D-biotin (blocking dose). L = liver, K = kidney, B = bladder.

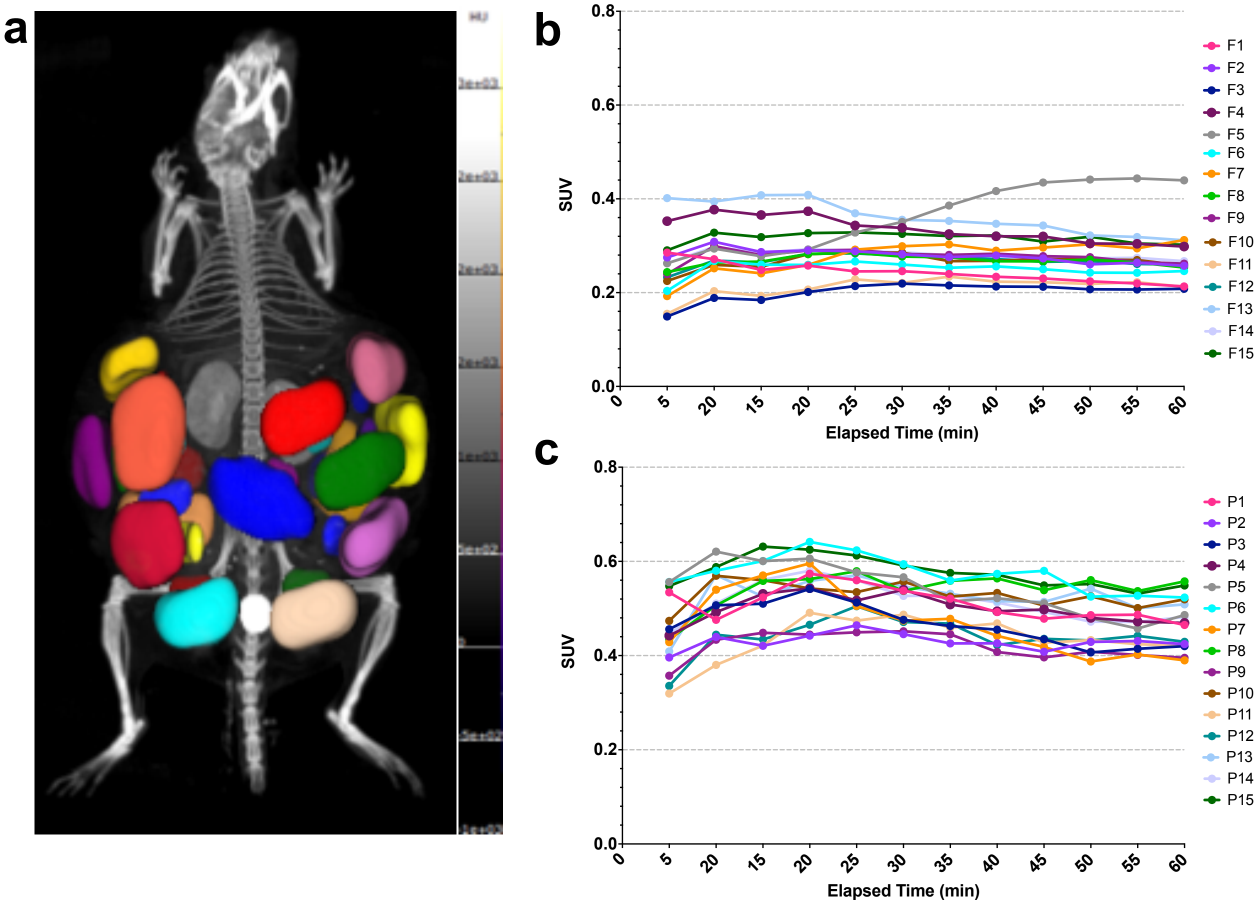

Figure S9. **(a)** is a maximal intensity projection (MIP) of just the CT of the same non-blocking mouse represented in **Figure 6b**. The regions of interest (ROIs) for each fetus (large) and placentae (small) are depicted as 3D shapes around the mid section of the mouse. **(b)** is a representative graph of the time activity curve, standard uptake value (SUV) over time, for each fetus in the mouse. **(c)** is a representative graph of the time activity curve for each placenta in the mouse.

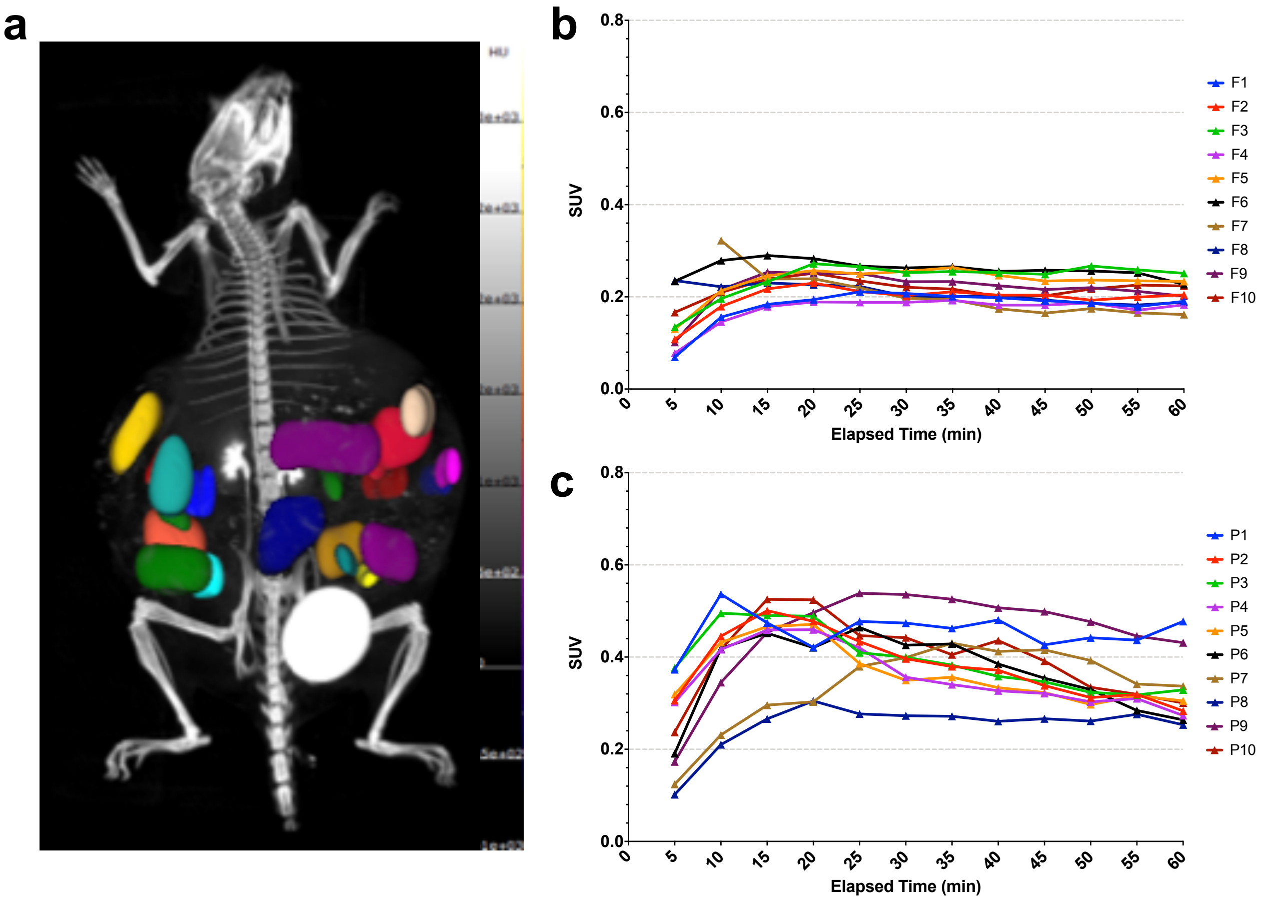

Figure S10. (a) is a maximal intensity projection (MIP) of just the CT of the same blocking mouse represented in **Figure 6c**. The regions of interest (ROIs) for each fetus (large) and placentae (small) are depicted as 3D shapes around the mid section of the mouse. **(b)** is a representative graph of the time activity curve, standard uptake value (SUV) over time, for each fetus in the mouse. **(c)** is a representative graph of the time activity curve for each placenta in the mouse.

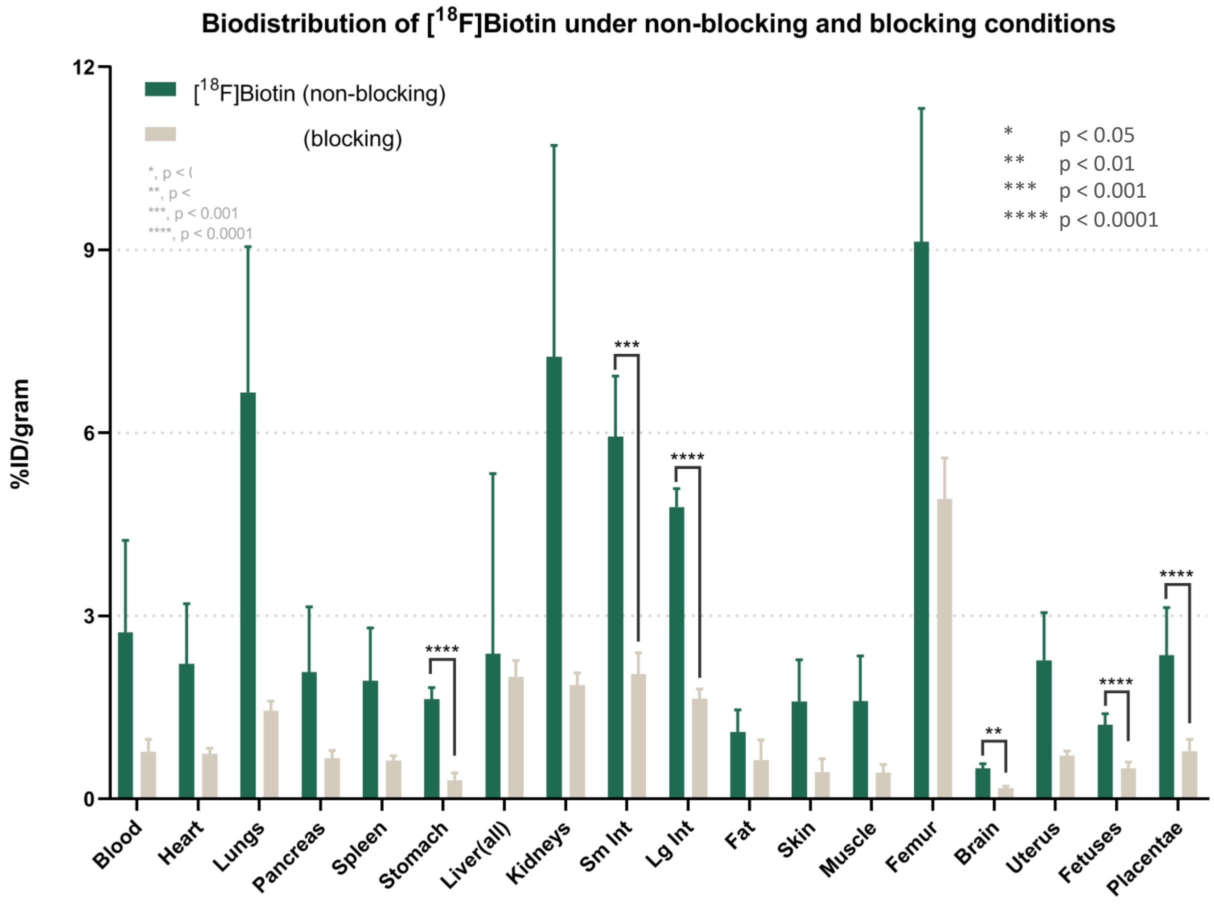

Figure S11. Whole body biodistribution of [^{18}F]Biotin under non-blocking (green) and blocking (tan) conditions one hour post injection.
